# Amyloid-Like Aggregation in Diseases and Biomaterials: Osmosis of Structural Information

**DOI:** 10.3389/fbioe.2021.641372

**Published:** 2021-03-03

**Authors:** Nicole Balasco, Carlo Diaferia, Giancarlo Morelli, Luigi Vitagliano, Antonella Accardo

**Affiliations:** ^1^Institute of Biostructures and Bioimaging (IBB), CNR, Naples, Italy; ^2^Department of Pharmacy, Research Centre on Bioactive Peptides (CIRPeB), University of Naples “Federico II”, Naples, Italy

**Keywords:** amyloid aggregates, cross-β structure, peptide-based hydrogels, amino acid aggregation, glutamine rich structures, biomaterials

## Abstract

The discovery that the polypeptide chain has a remarkable and intrinsic propensity to form amyloid-like aggregates endowed with an extraordinary stability is one of the most relevant breakthroughs of the last decades in both protein/peptide chemistry and structural biology. This observation has fundamental implications, as the formation of these assemblies is systematically associated with the insurgence of severe neurodegenerative diseases. Although the ability of proteins to form aggregates rich in cross-β structure has been highlighted by recent studies of structural biology, the determination of the underlying atomic models has required immense efforts and inventiveness. Interestingly, the progressive molecular and structural characterization of these assemblies has opened new perspectives in apparently unrelated fields. Indeed, the self-assembling through the cross-β structure has been exploited to generate innovative biomaterials endowed with promising mechanical and spectroscopic properties. Therefore, this structural motif has become the *fil rouge* connecting these diversified research areas. In the present review, we report a chronological recapitulation, also performing a survey of the structural content of the Protein Data Bank, of the milestones achieved over the years in the characterization of cross-β assemblies involved in the insurgence of neurodegenerative diseases. A particular emphasis is given to the very recent successful elucidation of amyloid-like aggregates characterized by remarkable molecular and structural complexities. We also review the state of the art of the structural characterization of cross-β based biomaterials by highlighting the benefits of the osmosis of information between these two research areas. Finally, we underline the new promising perspectives that recent successful characterizations of disease-related amyloid-like assemblies can open in the biomaterial field.

## Background and Introduction

The ability of the carbon atom to combine easily with other elements of the periodic table through covalent bonds is universally considered as a central factor for the development of life and of its related molecular diversity and complexity (Pace, 2001). The ability of this atom to form long chains is, however, only one of the founding aspects of life. Indeed, equally important is the capability of the building blocks of the molecules and macromolecules of life to establish a variety of non-covalent interactions that lead to the generation of the intricate architectures frequently exhibited by biomolecules and that regulate their mutual interactions. Whereas the definition of the skeleton of the covalent bonds of these molecules has progressively become a rather straightforward task despite its chemical complexity, the understanding of the bases that govern non-covalent interactions is still a matter of intense and, not rarely, unproductive work. Although many significant progresses have been recently achieved, the decrypting of the folding code of macromolecules or the prediction of intermolecular partnerships remain extremely challenging tasks (Baker, 2019; Senior et al., 2020; Lensink et al., 2020).

This intricate scenario has been further enriched by the discovery that the polypeptide chain has a remarkable and intrinsic propensity to form non-covalent aggregates, denoted as amyloid for their macroscopic reminiscence to starch-like deposits (Riek and Eisenberg, 2016; Chiti and Dobson, 2017). The interest for these particular supramolecular assemblies has been initially dictated by the discovery that in several neurodegenerative diseases, such as Parkinson and Alzheimer diseases and Huntington chorea, proteins and peptides, generally showing completely unrelated sequences, undergo structural transitions (misfolding) (Chiti and Dobson, 2017; Soto and Pritzkow, 2018). In contrast to what observed in physiological conditions where proteins are generally biochemical recyclable and degradable entities, in amyloidosis diseases, upon misfolding, specific proteins become resistant to the normal desegregation and turnover. Consequently, progressive accumulation and extracellular deposition occur in the different tissues, including brain, kidney and heart. The characterization of this proteinaceous material has unraveled a supramolecular organization in which synergic non-covalent interactions generally lead to the formation of extremely stable and low soluble structures (Soto, 2003; Chiti and Dobson, 2017). Beyond oligomers, annular and short/quiescent fibrils and protofibrils, other important forms of amyloid organization comprise superstructures, including spherulites, amyloid-crystals and microparticles (Krebs et al., 2004; Cannon and Donald, 2013; Reynolds et al., 2017).

**FIGURE 1:**
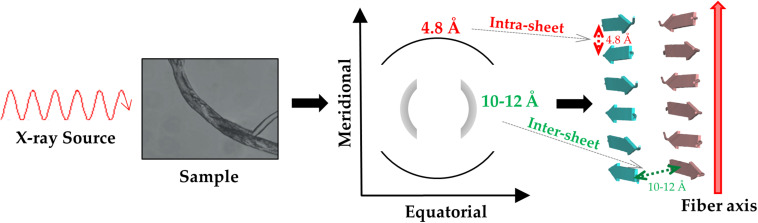
Schematic representation of the diffraction pattern and of the structure of a typical cross-β assembly.

The structural characterization of these misfolded protein states has progressively shown that both the main chain and the side chain atoms that concur to the formation of non-covalent interactions stabilize them. More specifically, the backbone moieties that participate to hydrogen bonds as hydrogen donors (CO) or acceptors (NH) generate a network of H-bonds that associates β-strands in β-sheet secondary structure. In the case of misfolded states, however, the hydrogen bond pattern is perpendicular to the growth axis of the assembly to generate the so-called cross-β structure (Figure 1; Sunde et al., 1997; Eisenberg et al., 2006). This structural motif presents a characteristic fiber diffraction pattern that exhibits a strong meridional reflection at 4.7–4.8 Å and a broad equatorial reflection in the resolution range 10–12 Å that correspond to the inter-strand and inter-sheet distances, respectively (Figure 1). Side chains contribute to the stability of these assemblies by making a variety of polar and apolar interactions that stabilize the structure of the individual β-sheets (ladder interactions) and the inter-sheet interfaces (Tsai et al., 2005).

Interestingly, the progressive molecular and structural characterization of these assemblies suggested that they could be also formed by rather small and very simple peptides (Gazit, 2007; Wang et al., 2018). This important discovery has led to the growth of a new area of research devoted to the development of innovative and bioinspired biomaterials based on small peptides assembling in cross-β structures. These biomaterials have shown excellent, and often unexpected, mechanical and spectroscopic properties that make them suitable for a myriad of applications in different biomedical and biotechnological areas (Gras, 2007; Maity et al., 2014; Kemper et al., 2015; Peralta et al., 2015; Tena-Solsona et al., 2016; Avitabile et al., 2018; Hu et al., 2018; Diaferia et al., 2019a).

Here, we analyze the state of the art about structural characterizations of cross β-sheet biomaterials and misfolded aggregates of proteins/peptides involved in neurodegenerative diseases. Although these self-assembling biomolecules share a common basic element, the methodologies used and the level of accuracy achieved for their structural characterizations present significant differences. It is important to stress that many of the topics here reported have been already illustrated in excellent literature reviews (see for example Cavalli et al., 2010; Riek and Eisenberg, 2016; Wei et al., 2017). However, it is also worth noting that new exciting results and ideas come out in literature almost daily. The present review is aimed at providing a general view of the state of the art of structural characterizations of cross β-sheet assemblies with a specific focus on the mutual benefits that each of these fields has gained from the success of the other. A particular emphasis is given to the impact that the impressive breakthroughs achieved in the structural characterization of misfolded proteins involved in neurodegeneration in the very last years could have on the possible development of innovative biomaterials.

## The Temporal Evolution of the Structural Characterization of Amyloid-Like Aggregates Involved in Disease

**FIGURE 2:**
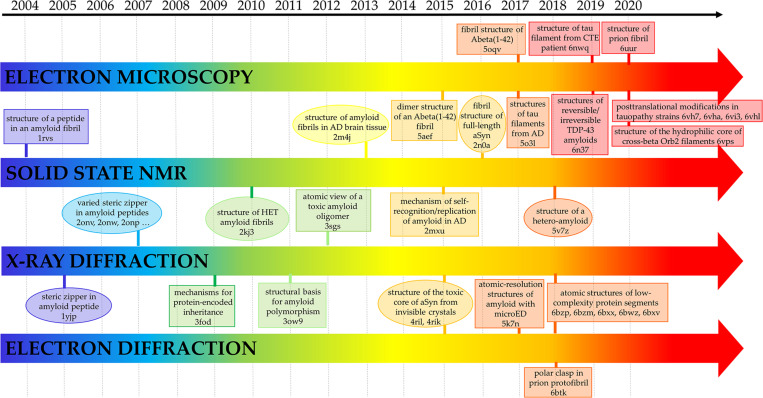
Selected milestones in the atomic-level characterization of cross-β structures related to diseases. The Protein Data Bank code of the related structures is also reported. AD, aSyn, CTE, microED are the abbreviations for Alzheimer Disease, α-Synuclein, Chronic Traumatic Encephalopathy and micro Electro Diffraction, respectively.

Some of the first insightful experiments in structural biology were performed by Astbury in mid 30ties who obtained the fiber diffraction patterns of different protein samples (Astbury and Street, 1931; Astbury et al., 1935). Although he was unable to derive atomic-level models from these data, he obtained meaningful diffraction patterns for the most common motifs observed in protein structures, i.e., α- and β-structure. Astbury was also able to register data from denatured samples of proteins displaying a pattern that was similar but rotated to that associated to the β-structure (cross-β structure). For years, the diffraction data of the α- and β-structure were extensively investigated with the aim of deriving the underlying atomic models. These were eventually obtained by Linus Pauling who theoretically built the correct structures for the α-helix and the β-sheet (Pauling et al., 1951; Pauling and Corey, 1951). On the other hand, the data and the possible related structure of the cross-β structure was somehow overlooked. The interest for this structural motif raised when its characteristic diffraction pattern was detected for misfolded proteins involved in neurodegeneration. Although the basic features of the cross-β structure could be straightforwardly derived from the diffraction pattern (Figure 1), the determination of the atomic structures of proteins/peptides adopting this motif has been a difficult process (Balbirnie et al., 2001; Eisenberg et al., 2006). As illustrated in Figure 2, this field has taken advantage of different experimental techniques that, in a step-by-step process started 15 years ago, have progressively led to the structural characterization of misfolded systems of increasing biological relevance. Figure 2 also shows that the most important contributions to the field come from X-ray crystallography (Eisenberg and Sawaya, 2017) and from cryo Electron Microscopy (cryoEM) (Fitzpatrick and Saibil, 2019) with interesting results also obtained by using solid state NMR (ssNMR) (Tycko, 2011) and Electron Diffraction (ED) studies conducted on extremely tiny crystals (microED) (Nannenga and Gonen, 2019). The remarkable contribution of computational studies in unraveling the structural/dynamic properties of self-assembling systems involved in pathogenic processes has been reviewed elsewhere (Nasica-Labouze et al., 2015). These three-dimensional structural data have been also exploited to design inhibitors of amyloid-like aggregation (Seidler et al., 2018; Griner et al., 2019) and nanovaccines (Luo and Abrahams, 2014; Al-Halifa et al., 2020; Zottig et al., 2020). It is also important to note that solution studies conducted with Small-Angle X-ray Scattering (SAXS) and Small-Angle Neutron Scattering (SANS) techniques have provided a considerable contribution to the characterization of solvent structure and dynamics of cross-β assemblies (Fichou et al., 2015; Langkilde et al., 2015; Pounot et al., 2020).

Interestingly, X-ray crystallography dominated the field in the early stage of this process (Figure 2). Indeed, very important results were achieved using a reductionist approach that consisted in the identification and the characterization of small peptide fragments that could be crystallized and that in some way could mimic the aggregation tendency and the properties of the misfolded parent protein/polypeptide (Eisenberg et al., 2006). Obviously, the major limitation of the technique is related to the crystallization of the biological relevant misfolded biomolecules whose size and tendency to form fibers make the crystallization process difficult if not impossible. In recent years, this scenario has drastically changed due to the impressive progresses made in the structural characterization of biomacromolecules using single particle cryoEM, a technique that is revolutionizing this field and the entire structural biology. This is clearly evident from the inspection of the right side of Figure 2 that highlights some of the milestones achieved using cryoEM.

The first experimental structural model of an amyloid-like system was the peptide corresponding to the 105-115 fragment of the amyloidogenic protein transthyretin whose structure was determined by magic angle spinning NMR spectroscopy (Jaroniec et al., 2004). The experimental data demonstrated that the peptide adopts a β-strand conformation with the main- and side-chain torsion angles close to their optimal values observed in β-sheets. Although the structural data highlighted a degree of long-range order, generally associated only with crystalline materials, no information could be derived on the lateral association of different β-sheets.

**FIGURE 3:**
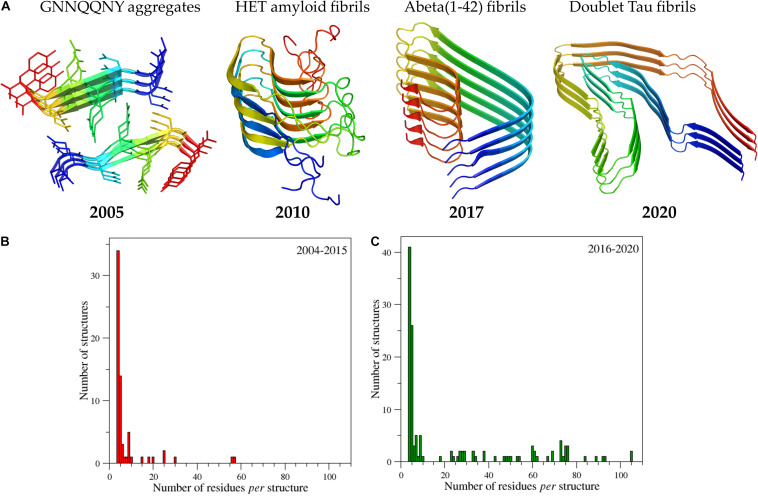
Progressive increase of structural complexity of the cross-β systems whose structure has been determined at atomic levels starting from the GNNQQNY peptide to the human brain-derived Tau filaments **(A)**. The PDB code of the structures reported from the left to the right of panel **(A)** are 1yjp, 2kj3, 5oqv, and 6vh7. Ribbons are represented with a color code (blue to red) from the N- to the C-terminus. The panels **(B)** and **(C)** report the number of amyloid structures as function of the size deposited in the time interval 2004-2015 and 2016-2020, respectively. The initial search for amyloid-like structures was performed by interrogating the entire PDB (release of October 2020) using as a query the following criteria: Polymer Entity Type = “Protein”; Experimental Method = X-ray diffraction, electron microscopy, electron crystallography, solid-state NMR, fiber diffraction, neutron diffraction, solution scattering; Additional Structure Keywords = “amyloid”; Polymer Entity Sequence Length > 5. The PDB structures identified by the server were then manually checked and their main features were reported in the Supplementary Table 1.

As mentioned above and highlighted by Figure 2, X-ray crystallography provided the most important contributions to the first characterizations of the amyloid-like structures at atomic level. Indeed, once solved the problem of handling and collecting data from the very small crystals that amyloid-like peptides tend to form due to their propensity to form fibers, a remarkable number of high resolution structures was obtained in very few years. The structure of the eptapeptide with the sequence GNNQQNY, which has become a sort of a prototype in the structural studies of amyloid-like peptides (Nelson et al., 2005), showed that the side chains of facing β-sheets interdigitated each other to form the so-called steric zipper motif (Figure 3A). The observed tight packing of the side chains in this motif straightforwardly explains the typical irreversibility of the formation of amyloid-like aggregates. It is worth mentioning that, although the role of the steric zipper motif in amyloid-like assemblies was experimentally discovered with the structure of GNNQQNY, a similar interdigitation of the side chains was proposed for poly-glutamine aggregates on the basis of fiber diffraction data and molecular modeling (Sikorski and Atkins, 2005).

Subsequent structural studies carried out on peptide fragments taken from different proteins demonstrated that steric zipper is a frequent motif adopted by the spine of amyloid-like fibers (Sawaya et al., 2007). These structures also unraveled that this motif can be adopted in several different arrangements depending on the nature of the β-sheet (parallel or antiparallel) and on the relative orientations of the facing β-sheets. Despite their limited size, the characterization of these peptides also provided interesting insights into the (mis)function of the aggregates. Particularly relevant in this context is the discovery of polymorphic crystal structures of prion and other amyloid fragments that suggested intriguing structural mechanisms for protein-encoded inheritance (Wiltzius et al., 2009). In particular, combining the information derived from these structures that are stabilized by interactions formed between identical chains (homotypic) with those emerged from ssNMR studies (Paravastu et al., 2008) that highlighted the possibility of forming interactions between distant regions of the protein (heterotypic), packing and segmental polymorphisms were suggested (Colletier et al., 2011; Laganowsky et al., 2012; Lu et al., 2013; Cao et al., 2019).

As indicated by the histograms reported in Figure 3 and data reported in Supplementary Table 1, which lists the amyloid-like structures we detected in a survey of the Protein Data Bank (PDB – release of October 2020) and classify them as function of the size of the cross-β core, the structural complexity of the characterized systems has enormously grown in the very last years. Apart from the structure of the HET amyloid fibrils (van Melckebeke et al., 2010), which actually adopt a different structural organization (β-helix), structures embodying significant fragments of the parent misfolded proteins were determined only since 2015. These achievements include the fibril structure of the Aβ peptide 1-42, of α-synuclein, of the protease resistant portion of the prion protein, of the Tau protein, and of functional amyloids such as the drosophila protein Orb2 (Schmidt et al., 2015; Xiao et al., 2015; Rodriguez et al., 2015; Tuttle et al., 2016; Gremer et al., 2017; Fitzpatrick et al., 2017; Mompean et al., 2018; Falcon et al., 2018a, b, 2019; Salinas et al., 2018, 2021; Hervas et al., 2020; Glynn et al., 2020; Röder et al., 2020; Schweighauser et al., 2020; Zhang et al., 2020).

**FIGURE 4:**
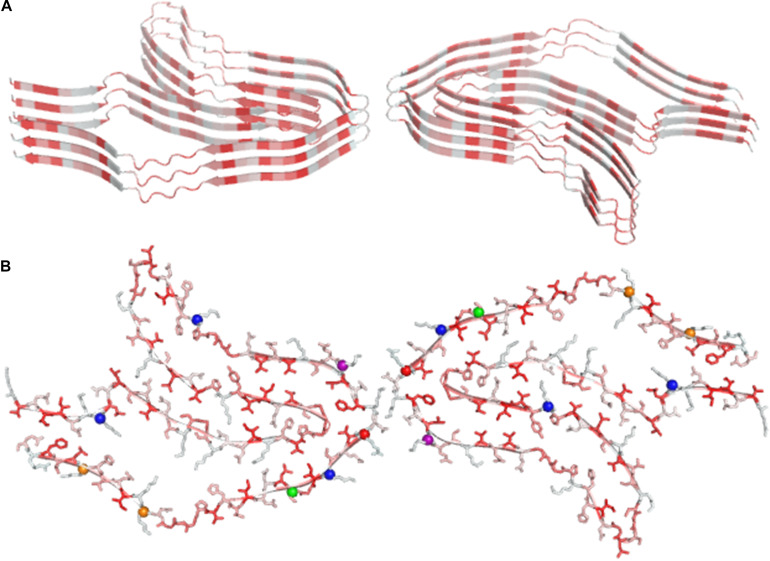
Ribbon **(A)** and ball and stick representation **(B)** of the doublet Tau Fibril from corticobasal degeneration human brain tissue, the largest cross-β structure currently deposited in the PDB (code 6vh7). Residues are colored as function of their hydrophobicity from gray (polar) to red (apolar). In panel B, posttranslational modification sites are denoted with balls.

**FIGURE 5:**
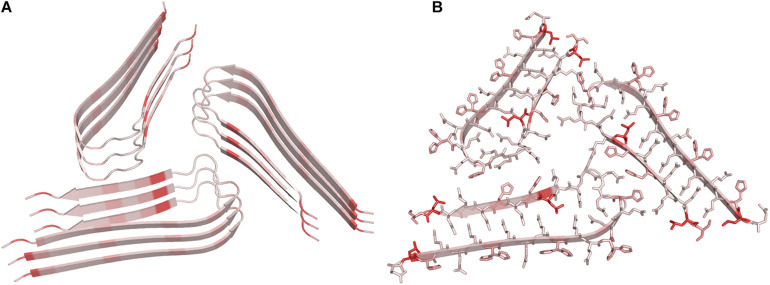
Ribbon **(A)** and ball and stick representation **(B)** structure of the hydrophilic core of cross-beta Orb2 filaments (PDB code 6vps) Residues are colored as function of their hydrophobicity from gray (polar) to red (apolar).

Remarkably, some of these structural studies have been performed using protein samples directly extracted from patients. According to our survey, the largest cross-β structure present in the PBD is the doublet Tau fibril from corticobasal degeneration human brain tissue (Arakhamia et al., 2020). In line with other large amyloid-like assemblies, this structure is stabilized by many heterotypic interactions that involve distant residues of the polypeptide sequence, in which hydrophobic contacts play a major role (Figure 4). Interestingly, large cross-β assemblies may also be stabilized by hydrophilic interactions as in the functional filaments of the protein Orb2 (Hervas et al., 2020), a putative substrate of long-lasting memories (Figure 5). In this functional amyloid, the side chains of the internal glutamine residues interdigitate to form a tight inter-sheet association that is virtually identical to that observed in the assemblies involved in the glutamine expansion diseases (Sambashivan et al., 2005; Sikorski and Atkins, 2005; Esposito et al., 2008; Colombo et al., 2008).

**FIGURE 6:**
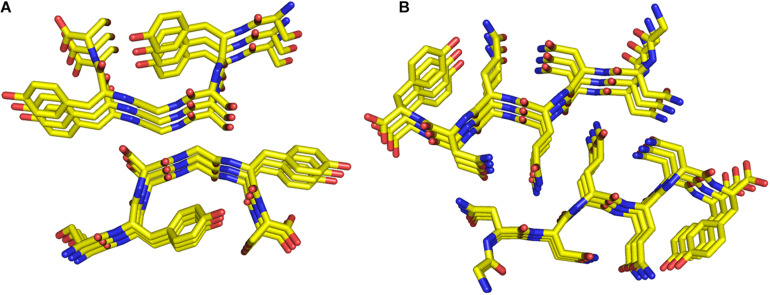
Ball and stick representation of the SYSGYS peptide (PDB code 6bwz) from the low-complexity domain of FUS **(A)**. The structure of the GNNQQNY peptide (PDB code 1yjp) is also reported **(B)**.

Finally, the structural characterization of small protein fragments by X-ray and electron diffraction crystallography has recently provided important contributions in the emerging area of biology aimed at characterizing subcellular membrane-less assemblies that form and re-dissolve in mammalian cells in response to stimuli (de la Cruz et al., 2017; Hughes et al., 2018; Gallagher-Jones et al., 2018). This type of phase separation is frequently observed with proteins that contain low-complexity domains (LCD) and that show a tendency to form reversible semi-solid phase hydrogels at high concentration. The structural characterization at atomic level of these proteins is extremely difficult. Moreover, although X-ray diffraction analysis of these hydrogels yields a cross-β pattern, fibrils found in these hydrogels are heat sensitive in contrast to amyloid fibrils that generally resist denaturation. The crystal structures of small LCD fragments isolated from a protein that forms these hydrogels (FUS) indicate that the heat sensitivity of these cross-β aggregates is related to the less tight association of the β-sheets inside the assemblies compared to steric zipper association detected in amyloids (Figure 6; Hughes et al., 2018).

## Going Small: Oligopeptide-Based Cross-β Assemblies for Biomaterials

As outlined in the previous section, initial attempts to gain molecular and structural information on misfolded states of proteins involved in neurodegenerative diseases were performed using a reductionist approach based on the identification of the minimal peptide fragments that were able to emulate the behavior of the parent protein/polypeptide. These studies were not only useful to get structural information on these systems, but they also suggested that very small peptides could be able to self-assemble.

**FIGURE 7:**
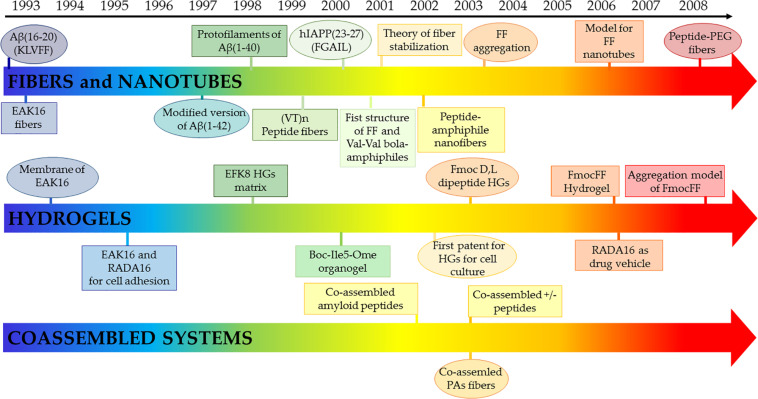
Selected early milestones on self-assembling peptides assuming cross-β structures as elements for fibers and nanotubes, hydrogels and co-assembled biomaterials. Reference for each group are reported following. *Fibers and nanotubes:* Aβ(16-20) (KLVFF) (Hilbich et al., 1992); EAK16 fibers (Zhang et al., 1993a); modified version of Aβ(1-42) (Seilheimer et al., 1997); protofilaments of Aβ(1-40) (Malinchik et al., 1998); (VT)n peptide fibers (Janek et al., 1999); hIAPP(23-27) (FGAIL) (Tenidis et al., 2000); Val-Val bola-amphiphiles (Kogiso et al., 2000); theory of fiber stabilization (Nyrkova et al., 2000); first crystallographic structure of FF (Görbitz, 2001); peptide-amphiphile nanofibers (Hartgerink et al., 2002); FF aggregation (Reches and Gazit, 2003); model for FF nanotubes (Görbitz, 2006); peptide-PEG fibers (Hamley and Krysmann, 2008). *Hydrogels:* membranes of EAK16 (Zhang et al., 1993b); EAK16 and RADA16 for cell adhesion (Zhang et al., 1993b); EFK8 HGs matrix (Leon et al., 1998); Boc-Ile5-Ome organogel (Jayakumar et al., 2000); Fmoc *D,L* dipeptide HGs (Zhang et al., 2003); FmocFF Hydrogel (Mahler et al., 2006); RADA16 as drug vehicle (Nagai et al., 2006); aggregation model of Fmoc-FF (Smith et al., 2008). *Co-assembled systems*: co-assembled amyloid peptides (Takahashi et al., 2002); co-assemled PAs fibers (Niece et al., 2003); co-assembled ± peptides (Aggeli et al., 2003).

Early successful examples of this reductionist approach include the identification of small amyloid-forming peptides from fragments of the islet amyloid polypeptide (the hexapeptide with sequence FGAILK) (Tenidis et al., 2000), human calcitonin (the pentapeptide DFNKFA) (Kazantzis et al., 2002) and the Aβ1-42 polypeptide (the hexapeptide KLVFFA) (Hilbich et al., 1992; Figure 7). In 2003, Reches and Gazit, extending the approach used to dissect the Aβ1-42 polypeptide and starting from the KLVFFA peptide, identified the diphenylalanine (FF) peptide as the shortest motif able to self-assemble (Görbitz, 2001; Reches and Gazit, 2003; Amdursky et al., 2010). Interestingly, due to its small size, minor modifications of the FF dipeptide lead to different supramolecular assemblies. Indeed, while H-FF-OH formed tubes, spheres, and quantum-dots, the FF variants H-FF-NH_2_ and Ac-FF-NH_2_ formed nanotubes (Yan et al., 2007; Adler-Abramovich and Gazit, 2014). Interestingly, carbobenzoxy-FF-OH, PEGylated-FF-OH, and Fmoc-FF-OH formed amyloid-like structures, nanofibers, and β-sheet-based fibrous-hydrogel, respectively (Reches and Gazit, 2005; Jayawarna et al., 2006; Mahler et al., 2006; Castelletto and Hamley, 2009; Diaferia et al., 2019b).

The seminal discovery on the ability of FF to self-assemble originated a new research area that developed in different directions (Yan et al., 2010). On one hand, it stimulated activities aimed at characterizing oligopeptides based on aromatic residues that are known to have a remarkable propensity to self-assemble. On the other hand, it also inspired studies focused on the self-assembling properties of single amino acids. The state of the art of these research lines will be outlined in the following paragraphs from a structural perspective.

Among oligopeptides based on aromatic residues, homopeptides formed by Phe residues, including tri-, tetra-, penta- and hexa-Phe peptides, have a special role. The analysis of the literature suggests that the length of the oligopeptide significantly influences structural, morphological and functional properties of the supramolecular architecture. In particular, the higher stability of these extended oligopeptides compared to the FF homodimer is due to the extended aromatic network in the peptide sequence. Opposite to FF dipeptide, FFF tripeptide forms planar nanostructures (Han et al., 2010), whereas its protected analogs Fmoc-FFF and Boc-FFF self-assemble into nanosphere (Chronopoulou et al., 2010) and hydrogels, respectively (Tamamis et al., 2008). Theoretical density functional theory (DFT) calculations carried out on the zwitterionic FFFF indicated the formation of irregular nanotubes resembling those achieved for FF. On the contrary, its Fmoc-FFFF derivative, lacking of the positive charge on the N-terminal end, can self-organize into a variety of assemblies (nanoplates, fibrils, star-like aggregates, and nanospheres), with a preference toward fibrillary structures with an antiparallel β-sheet organization. Instead, the double protected derivative Fmoc-FFFF-OBzl brought to the formation of volcano-like structures, triaxial ellipsoid-like nodules, and nanotubes (Mayans et al., 2015). Moreover, tetraphenylalanine derivatives in which the aromatic framework is derivatized with the Fmoc group (Fmoc-FFFF) or with polymeric chains such as polyethylene glycole, polycaprolactone, or polyethylene oxide keep a β-sheet structure (Castelletto and Hamley, 2009; Tzokova et al., 2009a, b; Diaferia et al., 2015, 2016a). Analogously to tetra-Phe also penta-Phe self-assembles into fibrillary structures in acetic acid, even though spectroscopic assays made by measuring Thioflavin T fluorescence are negative and the aggregates are not able to exhibit autofluorescence (Arnon et al., 2015).

Although biophysical characterizations of these assemblies provide some information on their structural preferences, models that illustrate their structures at atomic level are difficult to be experimentally obtained. Indeed, the intrinsic propensity of these systems to form fibers, which are generally twisted, disfavors the crystallization process. Moreover, their repetitive sequences make multiple associations between monomers energetically favorable, for example through alternative staggering of the pairing chains, which also have a negative impact on the growth of ordered crystals. Moreover, for very small peptides crystal packing may have a dramatic impact on their conformation. Therefore, crystalline states may not be fully representative of the real structure of these assemblies. Finally, the limited size of the basic spine of these assemblies makes the cryoEM technique hardly applicable.

**FIGURE 8:**
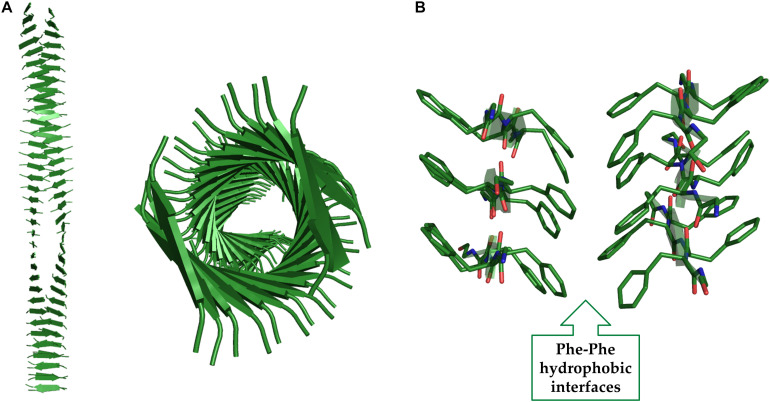
Two different views of the F_6_ structure obtained from MD studies **(A)**. The interactions at the hydrophobic interfaces are shown **(B)**.

A significant contribution to this field has been provided by the application of computational approaches (see for example López-Pérez et al., 2013; Do et al., 2015). Molecular modeling and molecular dynamics (MD) studies have indeed provided an atomic level description for F_6_ assemblies and demonstrated that Phe residues can tightly pack in anhydrous and rigid interfaces that stabilize the cross-β motif (Diaferia et al., 2016b, 2018a; Figure 8). These analyses have shown that Phe-side chains of facing β-sheets, although not interdigitated, establish repetitive and regular interactions that are quite common also in globular structures (Figure 8B). Similar arrangements into cross-β assemblies have been observed for other aromatic peptides based on either Tyr or Trp residues (e.g., Y_6_ and W_4_) (Diaferia et al., 2018b, c). MD simulations have also provided insights into the role that intra-sheet Phe-Phe interactions along with the terminal charged groups play in directing the associations of the β-strands, either parallel or antiparallel, within the β-sheets (Diaferia et al., 2018a). As mentioned above, the discovery of the FF self-assembling properties also stimulated the search for small, and even smaller than FF, aggregating systems. Limiting the size of the self-assembling entity has many synthetic advantages and reduces the costs of preparation of the resulting biomaterials. In this framework, many other natural occurring short and ultra-short peptide sequences demonstrated their tendency to self-assemble into ordered nanostructures (Frederix et al., 2015). For many years, it was assumed that dipeptides could represent the minimal entities able to allow generation of complex supramolecular structures due to the unique physicochemical properties of the amide bond. Only ten years after the identification of FF, Gazit and co-workers evaluated the possibility that, under specific conditions, also single amino acids could self-associate. This pioneering study was inspired by the evidence that many short amyloid-forming peptides contain non-consecutive phenylalanine residues (Gazit, 2002) as for example FGAIL and QRLANFLVH fragments of IAPP, NFGSVQFV of lactadherin protein, and SFNNGDCCFILD of gelsolin. In an independent analysis, Dobson and coworkers highlighted that aromatic amino acids, such as Phe and Trp, are the most amyloidogenic ones (Pawar et al., 2005). Although aromaticity is not an essential requirement for amyloid formation, it is clear that π-π interactions between aromatic moieties may accelerate the process of amyloid formation, especially for ultrashort peptide sequences in which the contribution of the hydrogen bonding is limited (Makin et al., 2005).

The first unmodified amino acid tested for its aggregating properties was Phe. The interest for the aggregation of this amino acid was also generated from the evidence that the congenital metabolic disorder phenylketonuria (PKU) is due to the accumulation of high levels of Phe in different portions of the brain, cerebrospinal fluid, and plasma (Kaufman, 1999). By using a series of biophysical techniques, which included electron microscopy, diffraction, thioflavin T and Congo red assays, it was demonstrated that at millimolar concentrations, phenylalanine could self-assemble into ordered nanofibrillar structures resembling amyloids (Adler-Abramovich et al., 2012). However, as detailed below, more recent studies have shown that structural characterization of Phe amyloid-like aggregation is not straightforward, thus demonstrating, once again, that the definition of the structure of assemblies formed by minimal peptide fragments may be a complicate task. In 2013, Perween et al. reported a further investigation on the aggregation properties of Phe in water solution under neutral pH. Although their analysis suggested that Phe self-assembles through different interactions such as hydrogen bonds and electrostatic interactions formed by the amino acid ends and π-π interactions established by the phenyl groups of adjacent molecules, no β-sheet structure was observed (Perween et al., 2013). The ability of the Phe amino acid to make multiple interactions was confirmed by the determination of a novel crystalline form of the zwitterionic L-phenylalanine (Mossou et al., 2014). In this monoclinic crystalline form, four Phe molecules are present in the asymmetric unit (Mossou et al., 2014). Phe layers are stabilized by alternating hydrophobic (aromatic environment) and hydrophilic interactions. Further information on the mechanism of assembly of phenylalanine oligomers and fibrils were obtained using ion-mobility mass spectrometry and theoretical calculations (Do et al., 2015). This study suggests that the Phe aggregation involves the formation of pore-like tetrameric oligomers. These layers of tetramers stack on top of each other to form an elongate structure and build multiple core structures through lateral π-stacking interactions.

Not surprisingly, later studies demonstrated the influence of the charged state of Phe ends on the morphology of the aggregates. In particular, in its zwitterionic state, Phe self-assembles into nanostructures with a fibrillary morphology, while in its cationic and anionic state, its aggregates are dominated by a flake morphology (Tomar et al., 2019). Moreover, several studies were also oriented to investigate different aggregation properties and kinetics of *L* and *D*-Phe. Both the isomers were found able to generate micrometer-long singular fiber structures, very similar to the amyloid-like assemblies. The crystal structure analysis of the pure Phe enantiomers highlighted that this aromatic amino acid self-organizes as consequence of hydrogen bonding and polar interactions between NH_3_^+^ and COO^–^ groups in neighboring molecules. On the other hand, the authors observed that both kinetics and final morphologies were significantly altered in *L/D* co-assembling samples (Bera et al., 2020). Specifically, the *DL*-Phe racemate showed inhibition of fiber organization with the formation of crystalline flake-like structural assemblies with a consequent difference also in the Young’s moduli (53.5 ± 12.1 GPa for *DL*-Phe composite system respect to 5.8 ± 0.7 and 1.8 ± 0.3 GPa for L- and D-isomer one, respectively). Dynamic simulations reveal that the chirality of amino acids and the lack of central symmetry in the crystals formed by these molecules promote crystal bending. The overall picture delineated above is further complicated by the discovery that Phe aggregates exhibit optical properties with a fluorescent emission peak at 485 nm that overlaps with the fluorescence emission spectra of the ThT/β-structure adduct. This result indicates that false-positive can occur in the ThT assay, which is generally used as a probe for amyloid structure. This evidence also suggests that this assay cannot be used to assess the amyloid nature of Phe aggregates (Ziaunys and Smirnovas, 2019).

Obviously, the possibility that other amino acids could exhibit self-assembling properties has been explored in the last decade. Exactly as for Phe, Tyr accumulation, which is due to the abnormal metabolic behavior of the tyrosine transaminase (TT) enzyme, was identified as the cause of tyrosinemia type II, another neurodegenerative disorder (Banik et al., 2017). The capability of Tyr to self-assemble was initially documented by Perween at al (Perween et al., 2013). They observed the formation of straight fibers with a flat ribbon-like texture from an aqueous solution of Tyr at a concentration of 1 mM, although the authors were not able to detect any typical signature of secondary structure adopted by the amino acid in these assemblies. No evidence for amyloid formation was found on different aggregates (fiber, dendrimeric structures and nanoribbon strip-like nanostructures) of Tyr obtained by varying its concentration (Mènard-Moyon et al., 2015). On the other hand, Gazit and coworkers pointed out the capability of Tyr to form amyloid resembling fibers, when aggregation occurs under different experimental conditions of pH (PBS), temperature (90°C), and concentration (∼22 mM) (Shaham-Niv et al., 2015). It seems, however, that the formation of Tyr amyloid fibers follows a different self-assembly process compared to that observed for Phe. Indeed, Banik et *al.* (Banik et al., 2017) demonstrated that in Tyr assemblies the H-bonding partner of the COO^–^ group is the OH present in its side chain whereas the carboxylate interacts with the NH_3_^+^ group in Phe aggregates. The self-assembling properties of unmodified tryptophan, which accumulates in pathological conditions related to two inborn errors of metabolism (hypertryptophanemia and Hartnup disease), were studied (Singh et al., 2017). On analogy with Tyr and Phe, this amino acid may form amyloid-like assemblies in specific conditions (at a concentration of ∼19.5 mM in phosphate buffer) (Shaham-Niv et al., 2017). On the other hand, in ethanol Trp self-assembles in nanotubes able to emit fluorescence when excited at 385 (blue), 488 (green), and 561 (red) nm (Babar and Sarkar, 2017). Several reports suggest that, despite the absence of π-π stacking interactions, also other amino acids like glycine, cysteine, methionine, and histidine can form ordered amyloid-like structures at millimolar concentrations (Gour et al., 2019). Structural studies highlighted that in these amino acids the fibril formation is prompted by hydrogen bonding occurring between the charged terminal groups NH_3_^+^ and COO^–^. This suggestion is supported by the observation that Cys and Met aggregates are amorphous in nature and can be obtained only under neutral pH conditions.

Finally, the growing evidences that pathological states of some inborn metabolic diseases could be correlated to the increase of endogenous metabolites prompted the researchers to evaluate the possibility that these metabolites could self-aggregate into amyloid-like structures (Shaham-Niv et al., 2015). In this scenario, Gazit and co-workers studied aggregation phenomena of some metabolites (adenine, orotic acid, cystine, tyrosine, uracil, and phenylalanine). Based on their observations, they suggest that the concept of amyloid aggregation could be also extended to non-peptidic chemical entities.

## Cross-β Hydrogels Formed by Proteins and Peptides

The β-structure in general and the cross-β motif in particular have also played a remarkable role in the development of peptide-based hydrogels (HG). HGs are self-supporting materials, structured as a supramolecular hydrophilic network associated with the construction of space-spanning structures characterized by a non-Newtonian behaviour (De Leon Rodriguez M. R. et al., 2016; Rivas et al., 2019). Their hydrophilic nature allows the entrapping of a high volume of biological fluids and water in the swelling process. Due to their appealing features, in the last years HGs have been exploited as versatile and innovative tools in nanomedicine as 3D-extracellular matrices for tissue engineering and regeneration, wound healing systems, ophthalmic compatible materials, and drug delivery systems (Yan and Pochan, 2010; Draper and Adams, 2017; Diaferia et al., 2019c). The specific 3D-connectivity of HGs may be generated by either a chemical irreversible junction (e.g., chemical bonds) or *via* non-covalent interactions (e.g., π-stacking, cation-π interaction, Van der Waals forces, and hydrogen bonding). Based on the different association modes of their basic components, HGs are broadly categorized as physically or chemically cross-linked materials. Moreover, based on their origin, HGs can be also classified as synthetic [e.g., poly(acrylic acid), poly(ε-caprolactone), poly(lactic acid), poly(glycolic acid)] or natural hydrogels (e.g., glycosaminoglycans, fibrin, chitosan, collagen) (Li et al., 2020).

### Protein-Based Hydrogels With Cross-β Structure

Proteins are attractive systems for generating HGs due to some specific properties that include biocompatibility, biodegradability, tunable mechanical properties, molecular binding abilities, and responses to external stimuli. They contain several functional and reactive groups that can be exploited for cross-linking. Interestingly, some proteins can form HGs without any chemical modification. Indeed, HGs may be occasionally formed by proteins in their misfolded states. Interesting examples include elastin (Flamia et al., 2007), α-synuclein (Bhak et al., 2010), lysozyme (Yan et al., 2006; Mains et al., 2013), Escherichia coli inner membrane protein YajC (YajC-CT) (Fang et al., 2011), and β-lactoglobulin (Gosal et al., 2004; Bolisetty et al., 2012), which were found able to self-aggregate, under specific conditions, into amyloid hydrogels of different nature in terms of stiffness, elasticity and biodegradability.

The low level of order of these HGs makes their atomic-level characterizations virtually unfeasible. A number of different biophysical techniques such as Thioflavin-T binding fluorescence, Congo red birefringence, Fourier Transform Infrared (FTIR), Transmission Electron Microscopy (TEM), Atomic Force Microscopy (AFM), cryo-Scanning Electron Microscopy (cryo-SEM) and Small-Angle Neutron Scattering (SANS) are generally used to assess their amyloid-like state. The molecular complexity of proteins and their articulated sequences make also difficult to identify their aggregation prone regions that constitute the spine of the HG. For elastin, some structural information on the aggregating region has been obtained through the characterization of the VGGVG pentapeptide and of the amyloids formed by poly(VGGVG) (Flamia et al., 2007).

### Peptide-Based Hydrogels With Cross-β Structure

The ability of peptides to self-assemble in cross-β structures has also been exploited for the development of innovative HGs (De Leon Rodriguez M. R. et al., 2016). Synthetic peptides exhibit a variable capability to gelificate depending on their amino acid sequence, hydrophilic/hydrophobic balance, net charge, and length. Experimental conditions such as peptide concentration, presence and concentration of electrolytes, the pH of the solution, and temperature can differently affect the functional and structural properties of the resulting hydrogel.

The gelation properties of peptides are also strongly affected by specific conjugations and modifications. A strategy that has been proposed for controlling mechanical and degradation properties of hydrogels, as well as to enhance their biocompatibility, is the functionalization of the peptide sequence with polymeric moieties. The advantage provided by the use of peptide-polymer conjugates is the chance to combine the features of each component into a unique building block (Redvar and Azevedo, 2019). Due to its high biocompatibility, water solubility and, long circulation time *in vivo*, poly(ethylene glycol) (PEG) has been exploited as polymer for derivatization of peptides to employ in the hydrogel formulation (Hamley, 2014). Peptide derivatization with PEG moiety was performed according to two different architectures of the conjugate: triblock (peptide-PEG-peptide) or diblock (PEG-peptide). However, it is worth noting that all peptides derivatized with PEG gelificate forming soft hydrogels with a storage modulus ranged between 10 and 100 Pa at a concentration < 5wt%. The gelation tendency of peptides may be deeply affected by their conjugation to aromatic groups such as Fmoc (fluorenylmethylcarbonyl) or naphthyl group. In this case, the hydrogelation process is favored by the additional stabilization effect (π–π stacking) due to the aromaticity of these molecules. Owing to this further stabilization effect, also very short peptide sequences containing either aromatic or non-aromatic amino acids were found to be able to gelificate. In extreme cases, the derivatization with the Fmoc group allows gelification of single amino acids (See also below). (Dasgupta et al., 2013; Fleming and Ulijn, 2014; Adler-Abramovich and Gazit, 2014).

Due to their simpler molecular complexity compared to proteins, the inspection of the sequences of peptides able to form HGs may provide insights into the structure of the final assemblies. In the most frequent cases, hydrogels are formed by peptides that present an alternation of amino acids in their sequences, typically polar/charged residues with apolar ones.

In addition to these peptides with alternating sequences, other HG forming peptides are made of stretches of hydrophobic and hydrophilic residues with the former assuring the formation of the cross-β structure and the latter contributing to the solubilization and to the interactions with the solvent. In the following paragraphs, HG-forming peptides are categorized and subcategorized using this conceptual framework. A specific section is dedicated to the HGs formed by very small peptides (dipeptides) and single amino acids (Table 1).

**TABLE 1:** Classification of peptide-based hydrogels assuming a cross-β structure with the relative reference.

HGs formed by peptides with alternating sequences	
**Alternation of apolar residues with both positively and negatively charged residues**	**Alternation of apolar residues with either positively or negatively charged residues**
Ac-(AEAEAKAK)_2_-NH_2_^[1]^	PE(LE)_5_P-OH^[12]^
Ac-RADARADARADARADA-NH_2_^[2]^	Ac-(IKIK)_2_-NH_2_^[13]^
Ac-RARADADARARADADA-NH_2_^[2]^	Ac-(FKFK)_2_-NH_2_^[13]^
Ac-(FKFE)_n_-NH_2_; n = 2,3,4^[3]^	Ac-(F5FKF5FK)_2_-NH_2_^[13]^
FEFEFKFK-OH ^[4]^	Ac-(ChaKChaK)_2_-NH_2_^[13]^
(FEFEFKFK)_2_-OH^[5]^	Ac-(VK)_5_-NH_2_^[13]^
Ac-(VKVE)_3_-NH_2_^[6]^	(VK)_4_V-NH_2_^[13]^
VKVKVEVK-OH^[7]^	(VK)_5_V-NH_2_^[13]^
VEVKVEVK-OH^[7]^	(VK)_6_V-NH_2_^[13]^
VEVEVEVK-OH^[7]^	(VR)_6_V-NH_2_^[14]^
Ac-(IKIE)_3_-NH_2_^[8]^	(IK)_6_I-NH_2_^[14]^
Ac-RLDLRLALRLDLR-NH_2_^[9]^	(LK)_6_L-NH_2_^[14]^
FFKLVFF-PEG2k^[10]^	Ac-(AAAK)_3_-NH_2_^[15–17]^
FFKLVFF-PEG3k^[10]^	Ac-(AAAK)_4_-NH_2_^[15–17]^
VEQLTEEQKNEFKAAFDIFVLGA-OH^[11]^	VKVKVKVKV^D^PPTKVKVKVKV-NH_2_^[18]^
**Alternation of apolar and uncharged polar residues**	**Gln-rich peptides**
PEG8-(FY)_3_-NH_2_^ [19]^	Ac-QQRQQQQQEQQ-NH_2_^[23]^
H-(Nal-DOPA)_3_-NH_2_^[20]^	Ac-QQRFQWQFEQQ-NH_2_^[23]^
Ac-K_2_(SL)_6_K_2_-NH_2_^[21]^	Ac-QQKFQFQFEQQ-NH_2_^[24]^
Ac-K_2_(QL)_6_K_2_-NH_2_^[21]^	
Ac-K_2_(TL)_6_K_2_-NH_2_^[21]^	
GSFSIQYTYHV-OH^[22]^	
**HGs formed by blocks of polar/apolar stretchs**	**HGs formed by conjugating single amino acids or dipeptides**
Ac-LIVAGD-OH^[25]^	Fmoc-F^[30]^
Ac-LIVAGDD-OH^[25]^	Fmoc-Y^[31]^
Ac-IVAGD-OH^[25]^	Fmoc-FF-OH^[32,33]^
Ac-IVD-OH^[25]^	Fmoc-FG-OH^[34]^
Ac-ILVAGD-OH^[25]^	Fmoc-DOPA-DOPA^[35]^
Ac-ILVAGS-OH^[25]^	Fmoc-F-Nphe^[36]^
Pal-VVVVAA-EEE-OH^[26]^	Fmoc-Nphe-F^[36]^
Pal-VVAAAA-EEE-OH^[26]^	Fmoc-Nphe-Nphe^[36]^
Pal-AAAVVV-EEE-OH^[26]^	Nvoc-FF^[37]^
Pal-VVVVAAAA-EEE-OH^[26]^	
Pal-VVVAAA-EEE-OH^[26]^	
Pal-VVAA-EEE-OH^[26]^	
Pal-VVVVAA-EEE-OH^[26]^	
Pal-VVAAAA-EEE-OH^[26]^	
Pal-AAAVVV-EEE-OH^[26]^	
Pal-GGGGGGG-ERGDS-OH^[27]^	
Pal-GGGGGGNMeG-ERGDS-OH^[27]^	
Pal-GGGGGNMeGG-ERGDS-OH^[27]^	
Pal-GGGGNMeGGG-ERGDS-OH^[27]^	
Pal-GGGGGNMeGNMeG-ERGDS-OH^[27]^	
Pal-V_3_K_2_-NH_2_^[28]^	
Lar-V_3_K_2_-NH_2_^[28]^	
Myr-V_3_K_2_-NH_2_^[28]^	
Myr-AA-OH^[28]^	
GSSAAAAAAAASGPGGYGPENQG PSGPGGYGPGGP^[29]^	

*[1] Zhang et al. (1993a,b), [2] Yokoi et al. (2005), [3] Swanekamp et al. (2014), [4] Saiani et al. (2009), [5]Wang et al. (2005), [6] Bowerman et al. (2011), [7] Roberts et al. (2012), [8] Caplan et al. (2002): [9] Nagai et al. (2012), [10] Castelletto et al. (2010), [11] De Leon-Rodriguez L. M. et al. (2016), [12] Rapaport et al. (2008), [13] Bowerman et al. (2011), [14] Geisler and Schneider (2012), [15] Measey and Schweitzer-Stenner (2006), [16] Measey et al. (2010), [17] Jang et al. (2009), [18] Nagy-Smith et al. (2015), [19] Diaferia et al. (2018b), [20] Diaferia et al. (2020), [21] Galler et al. (2010), [22] Frohm et al. (2015), [23] Davies et al. (2006): [24] Dawson et al. (1994), [25] Mishra et al. (2011), [26] Pashuck (2010): [27] Paramonov et al. (2006), [28] Wang et al. (2015), [29] Schacht and Scheibel (2011), [30] Singh et al. (2015), [31] Yang et al. (2021), [32] Mahler et al. (2006), [33] Jayawarna et al. (2006), [34] Tang et al. (2011), [35] Fichman et al. (2014a), [36]Rajbhandary and Nilsson (2017), [37] Roth-Konforti et al. (2018).*

#### Hydrogels Formed by Peptides With Alternating Sequences

In the β-structure arrangement, peptide sequences with an alternation of apolar and polar/charged residues direct their side chain in opposite directions, thus forming a hydrophobic and a hydrophilic face. The tight and rigid association of the two apolar faces generates the basic cross-β motif that constitutes the spine of the filaments within the HG. On the other hand, the hydrophilic interfaces interact with the solvent and mediate the interaction of different filaments in a non-regular way. However, it is worth noting that the nature of the polar/charged residues can strongly affect the possible interactions between the solvent and the exposed faces. The polar/charged residues may also stabilize the cross-β structure by making inter-sheet interactions with residues of adjacent strands. This class may be further subdivided based on the type of polar/charged residues present in the sequence. Indeed, the nature of these residues strongly affects the possible interactions between the solvent and the exposed faces. Since, within the same sheet, these residues can interact with those of adjacent strands, their presence also influences the type (parallel or antiparallel) of β-structure. It is likely that the simultaneous presence of both positively and negatively charged residues in the sequence may stabilize the cross-β motif and the formation of antiparallel β-sheets. On the other hand, the presence of only negatively or positively charged residues in the sequence may interfere with the association of the cross-β filaments in the HG. These evidences are detailed in the two next sections.

#### Alternation of Apolar Residues With Both Positively and Negatively Charged Residues

The prototype of this class of self-assembling peptides is EAK [Ac-(AEAEAKAK)_2_-NH_2_], which was designed and characterized by Zhang and co-authors (Zhang et al., 1993a). The sequence of this peptide exhibits an alternation of aliphatic (Ala) and ionic (Lys and Glu) residues. The dry interface of this peptide is built through the interdigitating of the methyl group of Ala side chains, a residue that has the well-defined ability to make this kind of assemblies. MD analyses indicate that this inter-sheet interface, which is approximately 6.5 Å wide, is rather rigid and confers a remarkable stability to the assembly (Calvanese et al., 2020). This study also indicates that interactions formed at the hydrophilic interface by the charged side chains contribute to the stabilization of the individual sheets present in the assembly. Over the years, EAK inspired the development of self-assembling peptides based on the same basic features i.e., the alternation in the sequence of Ala residues with positively or negatively charged residues. The peptides RADA16-I (Ac-RADARADARADARADA-NH_2_) and RAD16-II (Ac-RARADADARARADADA-NH_2_) are widely studied variants of EAK that share with the parent peptide the overall structural organization (Yokoi et al., 2005).

A similar conceptual approach has been used to develop new classes of self-assembling peptides by replacing Ala with other hydrophobic residues such as Phe (FEFEFKFK-OH, (FEFEFKFK)_2_, Ac-(FKFE)_2_-NH_2_, Ac-(FKFE)_3_-NH_2_, and Ac-(FKFE)_4_-NH_2_), Val (Ac-(VKVE)_3_-NH_2_, VKVKVEVK-OH, VEVKVEVK-OH, and VEVEVEVK-OH), Ile (Ac-(IKIE)_3_-NH_2_), and Leu (Ac-RLDLRLALRLDLR-NH_2_) (Table 1; Caplan et al., 2002; Wang et al., 2005; Saiani et al., 2009; Nagai et al., 2012; Roberts et al., 2012; Swanekamp et al., 2014). To this class, it can also be associated the peptide isolated from the protein troponin C (VEQLTEEQKNEFKAAFDIFVLGA-OH) (De Leon-Rodriguez L. M. et al., 2016). Although this peptide does not display a repetitive sequence, hydrophobic residues that can generate the hydrophobic interface can be identified in the sequence (H-KNEFKAAFDIFV-OH). These residues are surrounded by several charged ones that will be located on the hydrophilic face of the assembly.

#### Alternation of Apolar Residues With Either Positively or Negatively Charged Residues

A variation on the theme of the peptides displaying an alternation of hydrophobic residues with positively and negatively charged ones is represented by the class of peptides in which the apolar residues alternate exclusively with either negative or positive residues. The absence in these peptides of pair of residues that can form electrostatic interactions has an impact on both the stabilization of the individual sheets and on the lateral association of independent cross-β filaments.

Examples of peptides presenting only negative residues include the acidic β-sheet forming peptides (AAβP) that present the sequence PY(XY)_5_P, where Y is either Glu or Asp and X is either Phe or Leu. The pH value often plays a key role in the assembling of these alternating peptides into HGs. For example, the PE(LE)_5_P-OH peptide is able to form hydrogel only at a neutral pH value, where deprotonation of the acidic side chains occurs (Rapaport et al., 2008). As alternative, gelification of these negatively charged peptides was observed at low concentration in presence of Ca^2+^ ions. The coordination of this metal by the negatively charged side chains is an effective way to crosslink different cross-β filaments.

Positively charged β-sheet forming octapeptides with general sequence Ac-(XKXK)_2_-NH_2_, where X is Val, Ile, Phe, pentafluorophenylalanine (F5-Phe) or cyclohexylalanine (Cha), were able to gelificate in soft or hard hydrogels for non-aromatic or aromatic residue containing sequences, respectively. It is worth noting that gel formation by all these peptides, with the exception of Ac-(VKVK)_2_-NH_2_, occurs only at high values of ionic strength, where the electrostatic repulsions between the positive charges on lysine side chains are minimized (Bowerman et al., 2011). Lack of gelification in Ac-(VKVK)_2_-NH_2_ was attributed to the low hydrophobic interactions established by the valine residues. Indeed, it was observed that longer variants of this peptide such as (VK)nV-NH_2_ (with *n* = 4, 5, 6) and (VR)_6_V-NH_2_ were able to self-assemble into hydrogels (Geisler and Schneider, 2012). Successively, a systematic analysis of the aggregation properties of peptides obtained by replacing the valine residues in the sequence of the hydrogelator Ac-(VK)_n_V-NH_2_ with other aliphatic residues such as Ala, Ile or Leu highlighted the role of the hydrophobicity in the gelification process (Bowerman et al., 2011).

Later, other nine peptides presenting an alternation of hydrophobic (Leu and Ala) and hydrophilic positively charged (Lys and Arg) residues in their sequence were exploited as starting building blocks for the preparation of self-supporting hydrogels (Geisler and Schneider, 2012). Although these peptides exist in a random coil conformation in water, they undergo to aggregation phenomena upon addition of a buffered saline solution, by initially forming a β-sheet rich network of fibrils, ultimately leading to hydrogelation. Others two amphipathic variants of positively charged peptides (e.g., Ac-(AAAK)_3_-NH_2_ and Ac-(AAAK)_4_-NH_2_) that were able to form hydrogels at particularly low concentrations were then reported by Schweitzer-Stenner and coworkers (Measey and Schweitzer-Stenner, 2006; Jang et al., 2009; Measey et al., 2010).

The MAX1 peptide and its analogs also belong to this class (Dasgupta et al., 2013). MAX1 sequence (VKVKVKVK-V^D^PPT-KVKVKVKV-NH_2_) contains a tetrapeptide (V^D^PPT) assuming type II’ β-turn conformation in the middle of two extended strands with alternating hydrophobic (Val) and positively charged (Lys) residues (Table 1). Solid-state NMR characterization on self-assembled MAX1 in its fibrillary state pointed out that the peptide in the gel adopts a β-hairpin conformation and self-assembles into a double-layered cross-β structure (Nagy-Smith et al., 2015). Moreover, hairpins assemble with four probable structures differing in the nature of intermolecular alignments within and between the β-sheets.

#### Alternation of Apolar and Uncharged Polar Residues

**FIGURE 9:**
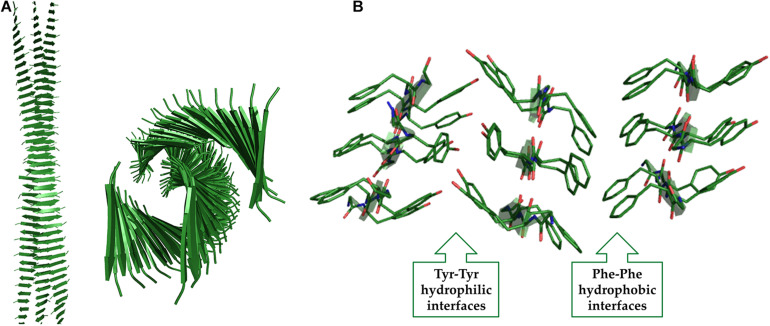
Two different views of the (FY)_3_ structure obtained from MD studies **(A)**. The interactions at the hydrophobic and hydrophilic interfaces are shown **(B)**.

The presence of charged residues in the peptides whose sequence presents an alternation of polar and apolar residues is not essential for aggregation and gelation. A remarkable example in this context is represented by the peptide H-(FY)_3_-NH_2_ (Diaferia et al., 2018b). This peptide presents a strong tendency to form fibrillar solid aggregates whose three-dimensional structure has been derived by combining Wide Angle X-ray Scattering (WAXS) data with molecular modeling and dynamics. The basic structural element of these assemblies formed by H-(FY)_3_-NH_2_ contain two distinct interfaces: a hydrophobic and highly rigid one made by the interactions of Phe residues and an hydrophilic one constituted by interacting Tyr side chains of facing strands (Figure 9). The conjugation of this peptide with the PEG moiety increases its solubility and leads to the formation of a hydrogel in which the Phe interface is likely retained in this state while the Tyr one is probably destabilized by the interaction with the solvent. In this conceptual framework it is not surprising that the peptide analogue H-(Nal-DOPA)_3_-NH_2_ (in which Nal is the 2-naphthylalanine and DOPA is the 3,4-dihydroxy-L-phenylalanine), designed as more hydrophilic variant of H-(FY)_3_-NH_2_ is able to form HGs without any type of PEG conjugation (Diaferia et al., 2020). The series of SL peptides (Ac-K_2_(SL)_6_K_2_-NH_2_, Ac-K_2_(QL)_6_K_2_-NH_2_ and Ac-K_2_(TL)_6_K_2_-NH_2_) also belongs to this type of HG-forming peptides, in which Leu and Ser residues make the hydrophobic and hydrophilic interfaces, respectively (Galler et al., 2010). Finally, an alternation of apolar/aromatic and non-charged polar residues may be identified in the natural peptide extracted from the semenolegin I protein (GSFSIQYTYHV-OH) that can be also associated to this class (Frohm et al., 2015).

Gln-rich peptides deserves a special description as they present some peculiar sequence features. Indeed, the capability of Gln to be part of both the internal and the external face of the cross-β motif (see for example Figure 6) makes these peptides particularly appealing. This ability is evident in the sequences of the cross-β peptides denoted as P11-I (Ac-QQRQQQQQEQQ-NH_2_) and P11-II (Ac-QQRFQWQFEQQ-NH_2_) in which Gln residues are frequently consecutive in the sequences (Davies et al., 2006). In P11-I the external face of the sheet includes the charged residues Arg and Glu that also favor the antiparallel orientation of the β-strands within the sheet. In P11-II, the cross-β motif is reinforced by replacing some internal Gln residues with other hydrophobic residues such as Phe and Trp. Above a critical concentration, P11 peptides are able to assemble into hydrogen-bonded β-sheet tapes. The further increase of concentration can lead to higher order structures compared to tapes. Indeed, since the amphiphilic tapes are endowed with both hydrophilic and hydrophobic faces, they can pair into ribbons which, in turn, can stack into fibrils and fibers through further intermolecular interactions (Collier and Messersmith, 2003). Based on P11–II, Collier and coworkers developed the β-sheet-forming peptide Q11 (Ac-QQKFQFQFEQQ-NH_2_), which can be covalently bound to bioactive molecules containing Lysine residues via tissue transglutaminase (Dawson et al., 1994). In this uncharged peptide the combination of the alternating FQFQF core and the short polyglutamine repeats allows the formation of stiff hydrogels.

#### Hydrogels Formed by Blocks of Polar/Apolar Stretchs

Another important class of peptides that can self-organize into β-sheet hydrogels is represented by amphiphilic peptides that present stretches of hydrophobic and hydrophilic residues. This class of peptides can be further sub-classified into: (i) surfactant like peptides (SLPs), and (ii) classical peptide amphiphiles (PAs). SLPs have a general formula X_*m*_Y_n_, where X is a hydrophobic amino acid (e.g., Gly, Ile, Leu, Val) and Y a polar charged amino acid (e.g., Asp, Glu, His, Lys, or Arg) and m and n are ranged between 3 to 8 and 1 to 2, respectively. Example of SLPs able to self-assemble into stiff hydrogels are Ac-LIVAGD-OH, Ac-LIVAGDD-OH, Ac-IVAGD-OH, Ac-IVD-OH, Ac-ILVAGD-OH, and Ac-ILVAGS-OH (Mishra et al., 2011). The rheological characterization of these gels highlighted that the length of the hydrophobic moiety and the polarity of the head group drastically affect the stiffness of the resultant hydrogel.

On the other hand, PAs contain a peptide sequence composed by a head group and a β-sheet inducer segment, which is in turn connected to a hydrophobic aliphatic tail of variable length. Nanofibers formation in PAs is allowed by the combination of hydrophobic interactions between the alkyl tails and hydrogen bonds between the side chains of the head group. A significant number of PA-based hydrogels have been described in literature for biomedical applications (De Leon Rodriguez M. R. et al., 2016; Machado et al., 2019; Diaferia et al., 2019d). Examples of PAs able to form hard hydrogels characterized by a cross-β sheet motif, with the general formula Pal-V_n_A_n_-EEE-OH (where *n* = 2, 3, 4), are Pal-VVVVAA-EEE-OH, Pal-VVAAAA-EEE-OH, and Pal-AAAVVV-EEE-OH, in which the hydrophobic tail is represented by the palmitoyl and the peptide sequence contains a variable number of Ala and Val residues and a tri-Glu head peptide, which promotes a stimuli-triggered peptide assembly (Pashuck et al., 2010). A similar structural transformation from twisted to helical ribbons was also observed in the PA containing three Phe residues (Pashuck and Stupp, 2010; Pashuck et al., 2010). As expected, in the series Pal-V_n_A_n_-EEE-OH, the hydrogel rigidity was found to be the highest for the longest peptide sequences, whereas the substitution of Val with Ala (as for example Pal-VVAAAA-EEE-OH compared to Pal-VVVVAA-EEE-OH) causes a dramatic decrease of the mechanical properties of the resulting hydrogels. This result is obviously due to the lower propensity of Ala residue to favor β-sheet assembly than Val. On the other hand, it was observed that the length of the aliphatic tail does not significantly affect the mechanical properties of PA-based hydrogels (Wang et al., 2015).

Other examples of PAs able to generate hydrogels are those containing the hepta-Gly peptide (Pal-GGGGGGG-ERGDS-OH) and its analogs in which one or more Gly residues in the sequence are replaced with NMeGly (N-Methyl-Glycine) (Paramonov et al., 2006). Due to the lower tendency of Gly to self-assemble into β-sheet, hepta-Gly peptides form soft hydrogels.

In 2011, Schacht et al. described another hydrogel based on the β-sheet forming peptide sequence from silk proteins, eADF4(C16) (GSSAAAAAAAASGPGGYGPENQGP SGPGGYGPGGP) containing 16 repeat units of module C. (Schacht and Scheibel, 2011). The eADF4 hydrogel formation occurs through a transformation from an initial random coil structure to α-helices, and eventually to β-sheets. The authors achieved an increase of stability and of mechanical stiffness in the hydrogels by chemically cross-linking tyrosine side chains.

#### Hydrogels Formed by Conjugating Single Amino Acids or Dipeptides

As mentioned above, the derivatization with Fmoc-group of small peptides and even single amino acids allows gelification (Dasgupta et al., 2013; Adler-Abramovich and Gazit, 2014; Fichman and Gazit, 2014; Fleming and Ulijn, 2014; Singh et al., 2015; Yang et al., 2021). Indeed, it is well known that the capability of small molecules to gelificate is strictly related to their hydrophobicity. Commonly, the total hydrophobicity is reported as logP, where P (repartition coefficient) is the ratio of concentrations at equilibrium of a compound in the two phases of a mixture made by two immiscible solvents. In line with this consideration, some of the Fmoc peptide derivatives have been deeply investigated as suitable building blocks for self-supporting hydrogels preparation. One of the most studied and promising dipeptide is Fmoc-FF-OH. The interest toward Fmoc-FF is to research into its capability of gelificate at physiological pH, compatible with biomedical applications (such as tissue engineering and drug delivery). Fmoc-FF firstly synthesized by Reches and Gazit (2005), self-assembles into macroscopic fibrous hydrogels above a critical concentration of 0.5%wt. Hydrogel formation is achieved either using the “solvent switch” method (Mahler et al., 2006), or as reported by Ulijn and co-workers, using the “pH-switch” method (Jayawarna et al., 2006). Structural studies on Fmoc-FF hydrogels allowed the proposal of a plausible theoretical aggregation model, in which peptide copies are arranged into a nanocylindrical structure (with a diameter of ∼3.0 nm) due to the interlocking through lateral π-π interactions of four twisted anti-parallel β-sheets. Under optimal pH conditions, lateral assembly of the nanostructures causes the formation of large flat ribbons. In the attempt to design novel simple peptide based materials, many others hydrogels based on Fmoc-dipeptides have been synthetized and characterized using both natural and unnatural amino acids. As for the long peptide sequences, it was observed that also for dipeptides both the number and the position of the aromatic residues in the peptide sequence played a key role in the self-assembling process and in the mechanical properties of the final material. In this context, the couple Fmoc-FG-OH and Fmoc-GF-OH represents an example in which the simple inversion between Phe and Gly (FG to GF) causes the loss of hydrogelation, thus indicating that the hydrogelation is favored for peptides containing adjacent aromatic groups (Tang et al., 2011). The importance of the primary peptide sequence in the hydrogel preparation was further evidenced by Adam’s group in their comparative study of the assembly process and of the mechanical properties of a small Fmoc-dipeptide library (Adams et al., 2010). Their results pointed out that only dipeptides with a logP value between 3.4 and 5.5 were able to form self-supporting hydrogels and the gel rigidity proportionally increases with the hydrophobicity. Moreover, the more hydrophilic Fmoc-FF variant, Fmoc-DOPA-DOPA, forms self-supporting hydrogels in which catechol groups are exposed to the solvent (Fichman et al., 2014b).

Successively, Rajbhandary and Nilsson synthetized three Fmoc-FF analogs (Fmoc-F-Nphe, Fmoc-Nphe-F and Fmoc-Nphe-Nphe) in which one or both the Phe residues were replaced with the N-benzyl glycine peptoid (Nphe) derivative, where the benzyl group in the amino acid is shifted from the C^α^ to the N^α^ atom (Rajbhandary and Nilsson, 2017). Their structural characterization indicated that only the two peptide/peptoid hybrids, keeping at least one Phe residue were able to gelificate, although with a lower propensity compared to Fmoc-FF. This result underlines the critical role played by the intermolecular H-bonding and the geometry of aromatic interactions in the gelation process. The effect of other aromatic groups on FF homodimer was also evaluated. For example, Adler-Abramovic et al. synthetized the Nvoc-FF (6-nitroveratryloxycarbonyl-diphenylylalanine), a Fmoc-FF analog in which the Fmoc group is replaced by the Nvoc one, a well-known ultraviolet (UV)-sensitive photo-trigger. This peptide forms light responsive hard hydrogels that can promote a controlled drug release upon UV irradiation (Roth-Konforti et al., 2018).

Analogously to Fmoc-dipeptides, also single amino acids derivatized with the Fmoc group have been found to be able to gelificate. In this case, hydrogelation is pH dependent and thermally reversible. The first example of fibrillation and subsequent hydrogelation by Fmoc protected single amino acids, alone or in combination, was reported by Xu and coworkers that described the hydrogelation of a mixture of Fmoc-Lys and Fmoc-Val upon basification (Yang et al., 2004). In order to exploit this small hydrogelator for industrial and biomedical applications, the hydrogelation of Fmoc-Phe and its aromatic analogues were also studied by other research groups (Sutton et al., 2009; [Bibr B191][Bibr B192]; Ryan et al., 2011). Fmoc-Phe gelificates by carefully adjusting the pH of the peptide solution from basic to neutral/acidic, whereas Fmoc-Tyr gelificates by diluting in water the peptide predissolved in an organic solvent at very high concentration. As alternative, the formation of self-assembled Fmoc-Y hydrogels can be promoted by a dephosphorylation reaction catalyzed by a phosphatase (Yang et al., 2008). Moreover, Shi et al. demonstrated that Phe can undergo to the gelification process also when functionalized with aromatic group alternative to Fmoc (e.g., naphthyl, naphthalenoxyl, or cinnamoyl) (Shi et al., 2011). The single crystal structure of Fmoc-F and Fmoc-Y gelators has been determined and the resulting data compared to the fiber X-ray diffraction data (Draper et al., 2015). Results indicated that there is a good match between data obtained on the fiber phase and on the crystalline phase of Fmoc-F. On the contrary, there are substantial differences between the two phases for Fmoc-Y. Indeed, in the crystal structure of Fmoc-Y the packing is mediated by π-π interactions between the aromatic rings of the Fmoc groups, whilst in the fiber the self-aggregation is prompted by hydrogen bonding within the gel. Analogously to Fmoc-F and Fmoc-Y also DOPA derivative self-assemble into hydrogels. Fmoc-DOPA is a non-coded analog of Fmoc-Y in which the catechol group replaces the phenol group of the tyrosine. Using a multistage and multiscale analysis, Fishman et *al.* proposed a model for the Fmoc-DOPA assembly mechanism, in which building blocks undergo to a progressive rearrangement from metastable spheres, in equilibrium with their monomeric form, up to thermodynamically favorable ultrastructures. The mechanism is based on three distinct stages: hydrophobic association in solution, self-assembly into ordered nanofibrils and crystallization due to the spatial locking and to the stabilization by hydrogen bonds (Fichman et al., 2016).

## Other Applications of Protein and Peptide Based Materials With β-Structure

With the advent of biosynthetic and efficient methods of purification, protein-based materials (recombinant, punctual chemically modified or engineered) are at the cutting-edge of materials science, electronics, and medicine (Vasconcelos et al., 2008; Abascal and Regan, 2018). Indeed, these materials are characterized by peculiar physical properties. Among these, their intrinsic biodegradability allows the process of circular economy that returns them to the total biomass. Moreover, as the cross-β motif represents a stable architecture in water-soluble polypeptides, it can be properly designed (Biancalana et al., 2010).

Different methodologies were developed to produce fibrils from protein-rich and renewable sources (*e.g.*, plant proteins) (Kamada et al., 2017; Josefsson et al., 2020). This aspect can explain the currently interest in both manufacturing and obtaining innovative protein-based systems. In this context, the first examples are related to natural fibers, including silk fibroin and resilin. Electrospunned into nanofibers, fibroin, corresponding to the protein of silk core, was proposed to prepare filaments for clothing (Aigner et al., 2018). This protein can be also wet-spun or self-organized to form microfibers, solvent cast into mesoporous foams for local dermal applications, used as enhancer for optoelectronic chips and nanorods or used as component of films. A solvent-based process can be used to form silk-based biodegradable screws or plates or to produce artificial skin and nanodots (Kishimoto et al., 2013; Saravanan et al., 2018; Maniglio et al., 2018; Cazares Vargas et al., 2019; Guo et al., 2020; Zheng and Zuo, 2021).

Resilin and elastin, together with their related materials, have been shaped properly for potential use in tissue engineering, in wound healing as porous scaffolds or films and as drug delivery systems. Aside from the reported applications, protein-based materials have shown potential use as conductive elements (Pena-Francesch et al., 2018). Protein- and peptide-based nanofibrils display interesting mechanical and functional properties in forming nanotubular scaffolding for bionanotechnology. For example, amyloid fibers were found to be mechanically strong (Lara et al., 2012; Bortolini et al., 2015; Paul et al., 2016), thus suggesting their potential use as constituents of functional systems and materials (Nuno et al., 2014; Knowles and Mezzenga, 2016). Moreover, they can be properly decorated by assembling antibody, fusion proteins or ligand to obtain biochemical sensors and chips (Men et al., 2010; Sakono et al., 2012; Kim et al., 2015; Oliveri et al., 2018). For instance, fibril aggregates have been proposed as potential tools for the next-generation of photovoltaic and organic solar cells (Barrau et al., 2008; Hauser et al., 2014), catalytic chemistry (Bolisetty et al., 2015; Al-Garawi et al., 2017) and energy-harvesting devices (Slabov et al., 2019). 2D amyloid hybrids fibrils were also used as immobilization platform for Au and enzymes (Pilkington et al., 2010; Fernández et al., 2016). These systems have also been applied as bioassay to clarify the mechanism of bacterial biofilm formation and of amyloid aggregation (Taglialegna et al., 2016; Taricska et al., 2020).

Peptide-based fibers were also proposed as innovative materials for biomedical applications, such as diagnostic agents, bioimaging agents and tissue engineering. For example, peptide nanofibers opportunely derivatized with gadolinium complexes were exploited as potential contrast agents for Magnetic Resonance Imaging (MRI) technique (Kim et al., 2016; Diaferia et al., 2019a; Gallo et al., 2020). On the other hand, the intrinsic blue/green photoluminescence recently associated to amyloid and amyloid-like structures has brought to the investigation of peptide and protein fibrils as fluorescent sources for monitoring the *in vitro* kinetics of aggregation (Liu et al., 2015; Gao et al., 2019). These photoluminescent materials (including peptide films and fibers), opportunely engineered, were evaluated as constituents for the development of integrated optoelectronic systems or waveguiding tools (Bo et al., 2019; Apter A. et al., 2020, Apter B. et al., 2020). Moreover, the chemical access, the biocompatibility and the high loading capacity of both hydrophobic and hydrophilic drugs displayed by these peptide and protein nanostructures make them appealing tools for drug delivery applications (Stie et al., 2020). Furthermore, fibrillary objects could be properly planned and decorated with bioactive motifs to achieve an active targeting of the drug. Stimuli-responsive, prolonged and triggered drug release profiles can also be obtained by modifying their primary sequence (Choi et al., 2016; Wei et al., 2017).

The unique physiochemical properties of amyloid fibrils make them attractive materials in a myriad of applications (Gras, 2007; Fichman et al., 2014a; Peralta et al., 2015), not just in biomedical and regenerative medical fields. Relevant applications were proposed in environmental sciences and liquid crystals (Corrigan et al., 2006; Castelletto and Hamley, 2009). For instance, amyloid fibrils, blended with activated carbon amyloid and forming macroscopic membranes, were proposed as purifying tools for a variety of contaminants from wastewater samples (Li and Mezzenga, 2012; Bolisetty and Mezzenga, 2016; Bolisetty et al., 2017). Modified lysozyme amyloid fibrils were also proposed as Chromium(VI) ions absorbent or as component for liquid crystal phases (Leung et al., 2016).

## Concluding Remarks and Future Perspectives

The intrinsic ability of the polypeptide chains to form β-rich structures, almost regardless of their length, has a tremendous impact in different research areas. As detailed in the previous sections, the interplay between the structural characterizations of small and large cross-β assemblies has been crucial to achieve an atomic level understanding of the aggregates involved in neurodegeneration and to develop innovative biomaterials. The reductionist approach has been fundamental for gaining the first information on structural bases of the aggregation modes of proteins. On the other hand, the structural characterization of protein/peptides assemblies in their misfolded states have provided important information for the definition of the atomic structures of self-assembling peptides.

This osmosis is likely to endure in the future, as the recent elucidation of the intricate structures of misfolded proteins will probably be exploited to create new biomaterials. In particular, the analysis of these structures reveals the presence of several heterotypic regions (see for example Figure 4) that could allow the design of couples of peptides whose mixing may generate novel self-assembling systems. Indeed, there is a growing interest toward hybrid hydrogels that can be generated by simply mixing two or more hydrogelators (Halperin-Sternfeld et al., 2017; Diaferia et al., 2019d; Zhai et al., 2019). This strategy may allow the creation of novel materials with improved mechanical properties, desired morphologies, implemented structural and functional complexity and enhanced stability. Moreover, the hydrophilic core of Orb2 filaments indicates that glutamine-based oligopeptides may represent an attractive source for the generation of innovative biomaterials due to the versatility of this residue that can be involved in both the anhydrous and the solvent exposed face of the sheet in the cross-β motif (Figure 5). Finally, new classes of peptide-based hydrogels may come from the characterization of peptides mimicking low-complexity domains of proteins involved in phase separation.

## Author Contributions

NB and CD performed the bibliographic research and arranged the graphical fashion. AA, GM, and LV conceptualized the topic and organized the manuscript. All the authors contributed to the writing steps and revisions.

## Conflict of Interest

The authors declare that the research was conducted in the absence of any commercial or financial relationships that could be construed as a potential conflict of interest.

## Supplementary Material

Supplementary Table 1 Reports the list of amyloid-like structure detected in a survey of the PDB (release of October 2020) including the PDB entry, the title, method, resolution, date of release and number of residues involved in cross-β core.
