# Supplementary material for: Amyloid-Like Aggregation in Diseases and Biomaterials: Osmosis of Structural Information
Source: Front Bioeng Biotechnol. 2021 Mar 3;9:641372. doi: 10.3389/fbioe.2021.641372 (PMC7966729; doi:10.3389/fbioe.2021.641372)
Supplement: Supplementary Table 1 — Reports the list of amyloid-like structure detected in a survey of the PDB (release of October 2020) including the PDB entry, the title, method, resolution, date of release and number of residues involved in cross-β core. [file Data_Sheet_1.DOCX]

| **PDB entry** | **Title** | **Method** | **Resolution** | **Released Date** | **Number of residues in the cross-β core** |
| --- | --- | --- | --- | --- | --- |
| 6WPQ | GNYNVF from hnRNPA2-low complexity domain segment, residues 286-291, D290V variant | X-RAY DIFFRACTION | 1.1 Å | 19/08/2020 | 4 |
| 6L1U | Cryo-EM structure of phosphorylated Tyr39 alpha-synuclein amyloid fibril | ELECTRON MICROSCOPY | 3.37 Å | 12/08/2020 | 84 |
| 6L1T | Cryo-EM structure of phosphorylated Tyr39 a-synuclein amyloid fibril | ELECTRON MICROSCOPY | 3.22 Å | 12/08/2020 | 93 |
| 6TI5 | A New Structural Model of Abeta(1-40) Fibrils | SOLID-STATE NMR | n.a. | 22/07/2020 | 28 |
| 6TI6 | Mixing Abeta(1-40) and Abeta(1-42) peptides generates unique amyloid fibrils | SOLID-STATE NMR | n.a. | 22/07/2020 | 29 |
| 6TI7 | Mixing Abeta(1-40) and Abeta(1-42) peptides generates unique amyloid fibrils | SOLID-STATE NMR | n.a. | 22/07/2020 | 29 |
| 6VW2 | Cryo-EM structure of human islet amyloid polypeptide (hIAPP, or amylin) fibrils | ELECTRON MICROSCOPY | 3.4 Å | 10/06/2020 | 18 |
| 6LNI | Cryo-EM structure of amyloid fibril formed by full-length human prion protein | ELECTRON MICROSCOPY | 2.702 Å | 10/06/2020 | 54 |
| 6PQ5 | AGAAAA segment 113-118 from human prion | X-RAY DIFFRACTION | 1.5 Å | 15/04/2020 | 4 |
| 6PQA | GAVVGG segment 119-124 from human prion | X-RAY DIFFRACTION | 1.46 Å | 15/04/2020 | 4 |
| 6UUR | Human prion protein fibril, M129 variant | ELECTRON MICROSCOPY | 3.5 Å | 15/04/2020 | 34 |
| 6VI3 | Straight Filament from Alzheimer's Disease Human Brain Tissue | ELECTRON MICROSCOPY | 3.3 Å | 15/04/2020 | 76 |
| 6LRQ | Cryo-EM structure of A53T alpha-synuclein amyloid fibril | ELECTRON MICROSCOPY | 3.49 Å | 08/04/2020 | 49 |
| 6RHA | Crystal structure of the amyloid-like NTVTFN segment from the Candida albicans Agglutinin-like protein (Adhesin) 5 | X-RAY DIFFRACTION | 1.6 Å | 25/03/2020 | 4 |
| 6RHB | Crystal structure of the amyloid-like IATLYV segment from the Candida albicans Agglutinin-like protein (Adhesin) 5 | X-RAY DIFFRACTION | 1.26 Å | 25/03/2020 | 4 |
| 6RHD | Crystal structure of the amyloid-like TSYVGV segment from the Candida albicans Agglutinin-like protein (Adhesin) 5 | X-RAY DIFFRACTION | 1.2 Å | 25/03/2020 | 4 |
| 6VPS | Cryo-EM structure of the amyloid core of Drosophila Orb2 isolated from head | ELECTRON MICROSCOPY | 2.6 Å | 18/03/2020 | 28 |
| 6Y1A | Amyloid fibril structure of islet amyloid polypeptide | ELECTRON MICROSCOPY | 4.2 Å | 04/03/2020 | 23 |
| 6VHL | Paired Helical Filament from Alzheimer's Disease Human Brain Tissue | ELECTRON MICROSCOPY | 3.3 Å | 04/03/2020 | 76 |
| 6VH7 | Doublet Tau Fibril from Corticobasal Degeneration Human Brain Tissue | ELECTRON MICROSCOPY | 3.8 Å | 04/03/2020 | 105 |
| 6VHA | Singlet Tau Fibril from Corticobasal Degeneration Human Brain Tissue | ELECTRON MICROSCOPY | 4.3 Å | 04/03/2020 | 105 |
| 6UFR | Structure of recombinantly assembled E46K alpha-synuclein fibrils | ELECTRON MICROSCOPY | 2.5 Å | 19/02/2020 | 61 |
| 6NK4 | KVQIINKKL, crystal structure of a tau protein fragment | ELECTRON CRYSTALLOGRAPHY | 1.994 Å | 15/01/2020 | 7 |
| 6SST | cryo-em structure of alpha-synuclein fibril polymorph 2B | ELECTRON MICROSCOPY | 3.4 Å | 18/12/2019 | 67 |
| 6SSX | cryo-em structure of alpha-synuclein fibril polymorph 2A | ELECTRON MICROSCOPY | 2.98 Å | 18/12/2019 | 69 |
| 6PEO | Cryo-EM structure of alpha-synuclein H50Q Narrow Fibril | ELECTRON MICROSCOPY | 3.3 Å | 27/11/2019 | 60 |
| 6PES | Cryo-EM structure of alpha-synuclein H50Q Wide Fibril | ELECTRON MICROSCOPY | 3.6 Å | 27/11/2019 | 60 |
| 6SDZ | transthyritin derived amyloid fibril from patient with hereditary V30M ATTR amyloidosis | ELECTRON MICROSCOPY | 2.97 Å | 13/11/2019 | 89 |
| 6SHS | Abeta fibril (Morphology I) | ELECTRON MICROSCOPY | 4.4 Å | 06/11/2019 | 33 |
| 6O4J | Amyloid Beta KLVFFAENVGS 16-26 D23N Iowa mutation | ELECTRON CRYSTALLOGRAPHY | 1.402 Å | 30/10/2019 | 5 |
| 6ODG | SVQIVY, Crystal Structure of a tau protein fragment | X-RAY DIFFRACTION | 1 Å | 02/10/2019 | 4 |
| 6R4R | Cryo-EM Structure of the PI3-Kinase SH3 Domain Amyloid Fibril | ELECTRON MICROSCOPY | 3.4 Å | 28/08/2019 | 75 |
| 6N37 | SegA-sym, conformation of TDP-43 low complexity domain segment A sym | ELECTRON MICROSCOPY | 3.8 Å | 26/06/2019 | 24 |
| 6N3C | SegB, conformation of TDP-43 low complexity domain segment A | ELECTRON MICROSCOPY | 3.3 Å | 26/06/2019 | 27 |
| 6N3B | SegA-asym, conformation of TDP-43 low complexity domain segment A asym | ELECTRON MICROSCOPY | 3.8 Å | 26/06/2019 | 33 |
| 6N3A | SegA-long, conformation of TDP-43 low complexity domain segment A long | ELECTRON MICROSCOPY | 3.3 Å | 26/06/2019 | 43 |
| 6RTB | cryo-em structure of alpha-synuclein fibril polymorph 2B | ELECTRON MICROSCOPY | 3.46 Å | 26/06/2019 | 62 |
| 6RT0 | cryo-em structure of alpha-synuclein fibril polymorph 2A | ELECTRON MICROSCOPY | 3.1 Å | 26/06/2019 | 69 |
| 6OC9 | S8 phosphorylated beta amyloid 40 fibrils | SOLID-STATE NMR | n.a. | 05/06/2019 | 23 |
| 6NZN | Dimer-of-dimer amyloid fibril structure of glucagon | SOLID-STATE NMR | n.a. | 05/06/2019 | 26 |
| 6G8C | Crystal Structure of the Amyloid-like IYQYGG segment from the R1 repeat of the E. coli Biofilm-associated CsgA Curli protein | X-RAY DIFFRACTION | 1.65 Å | 24/04/2019 | 4 |
| 6G8D | Crystal Structure of the Amyloid-like LNIYQY segment from the R1 repeat of the E. coli Biofilm-associated CsgA Curli protein | X-RAY DIFFRACTION | 1.85 Å | 24/04/2019 | 4 |
| 6G8E | Crystal Structure of the Amyloid-like VTQVGF segment from the R5 repeat of the E. coli Biofilm-associated CsgA Curli protein | X-RAY DIFFRACTION | 1.7 Å | 24/04/2019 | 4 |
| 6EEX | L-GSTSTA from degenerate octameric repeats in InaZ, residues 707-712 | X-RAY DIFFRACTION | 1.1 Å | 03/04/2019 | 4 |
| 6M7M | rac-GSTSTA from degenerate octameric repeats in InaZ, residues 707-712 | X-RAY DIFFRACTION | 1.101 Å | 03/04/2019 | 4 |
| 6IC3 | AL amyloid fibril from a lambda 1 light chain | ELECTRON MICROSCOPY | 3.3 Å | 03/04/2019 | 76 |
| 6M9I | L-GSTSTA from degenerate octameric repeats in InaZ, residues 707-712 | ELECTRON CRYSTALLOGRAPHY | 0.902 Å | 27/03/2019 | 4 |
| 6M9J | Racemic-GSTSTA from degenerate octameric repeats in InaZ, residues 707-712 | ELECTRON CRYSTALLOGRAPHY | 0.9 Å | 27/03/2019 | 4 |
| 6NWQ | Chronic traumatic encephalopathy Type II Tau filament | ELECTRON MICROSCOPY | 3.4 Å | 27/03/2019 | 53 |
| 6NWP | Chronic traumatic encephalopathy Type I Tau filament | ELECTRON MICROSCOPY | 2.3 Å | 27/03/2019 | 73 |
| 6HUD | Cryo-EM structure of cardiac amyloid fibrils from an immunoglobulin light chain (AL) amyloidosis patient. | ELECTRON MICROSCOPY | 4 Å | 27/03/2019 | 75 |
| 6MST | Cryo-EM structure of human AA amyloid fibril | ELECTRON MICROSCOPY | 2.7 Å | 13/03/2019 | 48 |
| 6GK3 | Two protofilament beta-2-microglobulin amyloid fibril | ELECTRON MICROSCOPY | 3.975 Å | 14/11/2018 | 60 |
| 6DIX | NFVFGT segment from Human Immunoglobulin Light-Chain Variable Domain, Residues 98-103, assembled as an amyloid fibril | X-RAY DIFFRACTION | 1 Å | 31/10/2018 | 4 |
| 6DJ0 | ASLTVS segment from Human Immunoglobulin Light-Chain Variable Domain, Residues 73-78, assembled as an amyloid fibril | X-RAY DIFFRACTION | 1.3 Å | 31/10/2018 | 4 |
| 6EKA | Solid-state MAS NMR structure of the HELLF prion amyloid fibrils | SOLID-STATE NMR | n.a. | 10/10/2018 | 47 |
| 6HRF | Straight filament from sporadic Alzheimer's disease brain | ELECTRON MICROSCOPY | 3.3 Å | 10/10/2018 | 74 |
| 6HRE | Paired helical filament from sporadic Alzheimer's disease brain | ELECTRON MICROSCOPY | 3.2 Å | 10/10/2018 | 75 |
| 6CU8 | Alpha Synuclein fibril formed by full length protein - Twister Polymorph | ELECTRON MICROSCOPY | 3.6 Å | 12/09/2018 | 38 |
| 6CU7 | Alpha Synuclein fibril formed by full length protein - Rod Polymorph | ELECTRON MICROSCOPY | 3.5 Å | 12/09/2018 | 50 |
| 6GX5 | Narrow Pick Filament from Pick's disease brain | ELECTRON MICROSCOPY | 3.2 Å | 12/09/2018 | 92 |
| 6FG4 | Crystal Structure of the Amyloid-like IIKVIK Segment from the S. aureus Biofilm-associated PSMalpha1 | X -RAY DIFFRACTION | 1.1 Å | 08/08/2018 | 4 |
| 6FGR | Crystal Structure of the Amyloid-like IIKIIK Segment from the S. aureus Biofilm-associated PSMalpha4 | X-RAY DIFFRACTION | 1.5 Å | 08/08/2018 | 4 |
| 6FHC | Crystal Structure of the Amyloid-like hexametric polymorph of the LFKFFK segment from the S. aureus PSMalpha3 . | X-RAY DIFFRACTION | 1.51 Å | 08/08/2018 | 4 |
| 6FHD | Crystal Structure of the Amyloid-like, out-of-register beta-sheets, polymorph of the LFKFFK segment from the S. aureus PSMalpha3 | X-RAY DIFFRACTION | 1.85 Å | 08/08/2018 | 4 |
| 5WOR | Corkscrew assembly of SOD1 residues 28-38 with familial mutation G37R | X-RAY DIFFRACTION | 2.77 Å | 30/05/2018 | 7 |
| 5WKB | MicroED structure of the segment, NFGEFS, from the A315E familial variant of the low complexity domain of TDP-43, residues 312-317 | ELECTRON CRYSTALLOGRAPHY | 1 Å | 23/05/2018 | 4 |
| 6CF4 | Segment NFGTFS, with familial mutation A315T and phosphorylated threonine, from the low complexity domain of TDP-43, residues 312-317 | ELECTRON CRYSTALLOGRAPHY | 0.75 Å | 23/05/2018 | 4 |
| 5WHP | Crystal structure of the segment, NFGTFS, from the A315T familial variant of the low complexity domain of TDP-43, residues 312-317 | X-RAY DIFFRACTION | 1 Å | 23/05/2018 | 4 |
| 6CFH | SWGMMGMLASQ segment from the low complexity domain of TDP-43 | ELECTRON CRYSTALLOGRAPHY | 1.5 Å | 23/05/2018 | 9 |
| 6CLC | 1.01 A MicroED structure of GSNQNNF at 0.27 e- / A^2 | ELECTRON CRYSTALLOGRAPHY | 1.01 Å | 16/05/2018 | 5 |
| 6CLD | 1.01 A MicroED structure of GSNQNNF at 0.81 e- / A^2 | ELECTRON CRYSTALLOGRAPHY | 1.01 Å | 16/05/2018 | 5 |
| 6CLE | 1.01 A MicroED structure of GSNQNNF at 1.3 e- / A^2 | ELECTRON CRYSTALLOGRAPHY | 1.01 Å | 16/05/2018 | 5 |
| 6CLF | 1.15 A MicroED structure of GSNQNNF at 1.9 e- / A^2 | ELECTRON CRYSTALLOGRAPHY | 1.15 Å | 16/05/2018 | 5 |
| 6CLG | 1.35 A MicroED structure of GSNQNNF at 2.4 e- / A^2 | ELECTRON CRYSTALLOGRAPHY | 1.35 Å | 16/05/2018 | 5 |
| 6CLH | 1.37 A MicroED structure of GSNQNNF at 2.9 e- / A^2 | ELECTRON CRYSTALLOGRAPHY | 1.37 Å | 16/05/2018 | 5 |
| 6CLI | 1.01 A MicroED structure of GSNQNNF at 0.17 e- / A^2 | ELECTRON CRYSTALLOGRAPHY | 1.01 Å | 16/05/2018 | 5 |
| 6CLJ | 1.01 A MicroED structure of GSNQNNF at 0.50 e- / A^2 | ELECTRON CRYSTALLOGRAPHY | 1.01 Å | 16/05/2018 | 5 |
| 6CLK | 1.01 A MicroED structure of GSNQNNF at 0.82 e- / A^2 | ELECTRON CRYSTALLOGRAPHY | 1.01 Å | 16/05/2018 | 5 |
| 6CLL | 1.02 A MicroED structure of GSNQNNF at 1.2 e- / A^2 | ELECTRON CRYSTALLOGRAPHY | 1.02 Å | 16/05/2018 | 5 |
| 6CLM | 1.01 A MicroED structure of GSNQNNF at 1.5 e- / A^2 | ELECTRON CRYSTALLOGRAPHY | 1.01 Å | 16/05/2018 | 5 |
| 6CLN | 1.15 A MicroED structure of GSNQNNF at 1.8 e- / A^2 | ELECTRON CRYSTALLOGRAPHY | 1.15 Å | 16/05/2018 | 5 |
| 6CLO | 1.15 A MicroED structure of GSNQNNF at 2.1 e- / A^2 | ELECTRON CRYSTALLOGRAPHY | 1.15 Å | 16/05/2018 | 5 |
| 6CLP | 1.16 A MicroED structure of GSNQNNF at 2.5 e- / A^2 | ELECTRON CRYSTALLOGRAPHY | 1.16 Å | 16/05/2018 | 5 |
| 6CLQ | 1.21 A MicroED structure of GSNQNNF at 2.8 e- / A^2 | ELECTRON CRYSTALLOGRAPHY | 1.21 Å | 16/05/2018 | 5 |
| 6CLR | 1.31 A MicroED structure of GSNQNNF at 3.1 e- / A^2 | ELECTRON CRYSTALLOGRAPHY | 1.31 Å | 16/05/2018 | 5 |
| 6CLS | 1.46 A MicroED structure of GSNQNNF at 3.4 e- / A^2 | ELECTRON CRYSTALLOGRAPHY | 1.46 Å | 16/05/2018 | 5 |
| 6CLT | 1.45 A MicroED structure of GSNQNNF at 3.8 e- / A^2 | ELECTRON CRYSTALLOGRAPHY | 1.45 Å | 16/05/2018 | 5 |
| 5WHN | Crystal structure of the segment, NFGAFS, from the low complexity domain of TDP-43, residues 312-317 | X-RAY DIFFRACTION | 1.1 Å | 25/04/2018 | 4 |
| 5WIA | Crystal structure of the segment, GNNSYS, from the low complexity domain of TDP-43, residues 370-375 | X-RAY DIFFRACTION | 1.002 Å | 25/04/2018 | 4 |
| 6C3F | AMYLOID FORMING PEPTIDE IYKVEI FROM TRANSTHYRETIN | X-RAY DIFFRACTION | 1.499 Å | 18/04/2018 | 4 |
| 6C3S | AMYLOID FORMING PEPTIDE YTIAAL FROM TRANSTHYRETIN | X-RAY DIFFRACTION | 1.602 Å | 18/04/2018 | 4 |
| 6C88 | STRUCTURE OF THE AMYLOID FORMING PEPTIDE VAVHVF FROM TRANSTHYRETIN | X-RAY DIFFRACTION | 1.851 Å | 18/04/2018 | 4 |
| 6CB9 | Segment AALQSS from the low complexity domain of TDP-43, residues 328-333 | X-RAY DIFFRACTION | 1.1 Å | 18/04/2018 | 4 |
| 6CEW | Segment AMMAAA from the low complexity domain of TDP-43, residues 321-326 | X-RAY DIFFRACTION | 1.2 Å | 18/04/2018 | 4 |
| 5WIQ | Crystal structure of the segment, GFNGGFG, from the low complexity domain of TDP-43, residues 396-402 | X-RAY DIFFRACTION | 1.25 Å | 18/04/2018 | 5 |
| 5WKD | Crystal structure of the segment, GNNQGSN, from the low complexity domain of TDP-43, residues 300-306 | X-RAY DIFFRACTION | 1.8 Å | 18/04/2018 | 5 |
| 6C4O | AMYLOID FORMING PEPTIDE TIAALLS FROM TRANSTHYRETIN | X-RAY DIFFRACTION | 1.79 Å | 18/04/2018 | 5 |
| 5XSG | Ultrahigh resolution structure of FUS (37-42) SYSGYS determined by MicroED | ELECTRON CRYSTALLOGRAPHY | 0.73 Å | 04/04/2018 | 4 |
| 6BZP | STGGYG from low-complexity domain of FUS, residues 77-82 | ELECTRON CRYSTALLOGRAPHY | 1.1 Å | 04/04/2018 | 4 |
| 5XRR | Crystal structure of FUS (54-59) SYSSYG | X-RAY DIFFRACTION | 1.503 Å | 04/04/2018 | 4 |
| 6BWZ | SYSGYS from low-complexity domain of FUS, residues 37-42 | X-RAY DIFFRACTION | 1.1 Å | 04/04/2018 | 4 |
| 6BXX | GYNGFG from low-complexity domain of hnRNPA1, residues 243-248 | X-RAY DIFFRACTION | 1.1 Å | 04/04/2018 | 4 |
| 6BZM | GFGNFGTS from low-complexity/FG repeat domain of Nup98, residues 116-123 | ELECTRON CRYSTALLOGRAPHY | 0.9 Å | 04/04/2018 | 6 |
| 6BXV | SYSSYGQS from low-complexity domain of FUS, residues 54-61 | X-RAY DIFFRACTION | 1.1 Å | 04/04/2018 | 6 |
| 5WMJ | KVWGSI segment from Superoxide Dismutase 1,residues 30-35 | X-RAY DIFFRACTION | 1.4 Å | 28/03/2018 | 4 |
| 5V7Z | SSNMR Structure of the Human RIP1/RIP3 Necrosome | SOLID-STATE NMR | n.a. | 28/03/2018 | 10 |
| 5W52 | MicroED structure of the segment, DLIIKGISVHI, from the RRM2 of TDP-43, residues 247-257 | ELECTRON CRYSTALLOGRAPHY | 1.4 Å | 21/02/2018 | 9 |
| 5V5C | VQIINK, Structure of the amyloid-spine from microtubule associated protein tau Repeat 2 | ELECTRON CRYSTALLOGRAPHY | 1.25 Å | 07/02/2018 | 4 |
| 5V5B | KVQIINKKLD, Structure of the amyloid spine from microtubule associated protein tau Repeat 2 | ELECTRON CRYSTALLOGRAPHY | 1.5 Å | 07/02/2018 | 8 |
| 6AXZ | Segment from bank vole prion protein 168-176 QYNNQNNFV | ELECTRON CRYSTALLOGRAPHY | 0.75 Å | 17/01/2018 | 7 |
| 6BTK | Segment from bank vole prion protein 168-176 QYNNQNNFV | X-RAY DIFFRACTION | 1.1 Å | 17/01/2018 | 7 |
| 5VOS | VGSNKGAIIGL from Amyloid Beta determined by MicroED | ELECTRON CRYSTALLOGRAPHY | 1.42 Å | 03/01/2018 | 9 |
| 5OQV | Near-atomic resolution fibril structure of complete amyloid-beta(1-42) by cryo-EM | ELECTRON MICROSCOPY | 4 Å | 13/09/2017 | 38 |
| 5O3L | Paired helical filament in Alzheimer's disease brain | ELECTRON MICROSCOPY | 3.4 Å | 26/07/2017 | 73 |
| 5O3O | Pronase-treated paired helical filament in Alzheimer's disease brain | ELECTRON MICROSCOPY | 3.5 Å | 26/07/2017 | 73 |
| 5O3T | Straight filament in Alzheimer's disease brain | ELECTRON MICROSCOPY | 3.4 Å | 26/07/2017 | 73 |
| 5IIW | Corkscrew assembly of SOD1 residues 28-38 without potassium iodide | X-RAY DIFFRACTION | 2 Å | 28/06/2017 | 7 |
| 5UGK | Zinc-Binding Structure of a Catalytic Amyloid from Solid-State NMR Spectroscopy | SOLID-STATE NMR | n.a. | 31/05/2017 | 5 |
| 5K7N | MicroED structure of tau VQIVYK peptide at 1.1 A resolution | ELECTRON CRYSTALLOGRAPHY | 1.1 Å | 05/04/2017 | 4 |
| 5KNZ | Human Islet Amyloid Polypeptide Segment 19-SGNNFGAILSS-29 with Early Onset S20G Mutation Determined by MicroED | ELECTRON CRYSTALLOGRAPHY | 1.902 Å | 21/12/2016 | 9 |
| 5KO0 | Human Islet Amyloid Polypeptide Segment 15-FLVHSSNNFGA-25 Determined by MicroED | ELECTRON CRYSTALLOGRAPHY | 1.4 Å | 21/12/2016 | 9 |
| 5K2E | Structure of NNQQNY from yeast prion Sup35 with zinc acetate determined by MicroED | ELECTRON CRYSTALLOGRAPHY | 1 Å | 14/09/2016 | 4 |
| 5K2F | Structure of NNQQNY from yeast prion Sup35 with cadmium acetate determined by MicroED | ELECTRON CRYSTALLOGRAPHY | 1 Å | 14/09/2016 | 4 |
| 5K2G | Structure of GNNQQNY from yeast prion Sup35 in space group P21 determined by MicroED | ELECTRON CRYSTALLOGRAPHY | 1.1 Å | 14/09/2016 | 5 |
| 5K2H | Structure of GNNQQNY from yeast prion Sup35 in space group P212121 determined by MicroED | ELECTRON CRYSTALLOGRAPHY | 1.05 Å | 14/09/2016 | 5 |
| 5DLI | Corkscrew assembly of SOD1 residues 28-38 | X-RAY DIFFRACTION | 2.1 Å | 14/09/2016 | 6 |
| 5KK3 | Atomic Resolution Structure of Monomorphic AB42 Amyloid Fibrils | SOLID-STATE NMR | n.a. | 13/07/2016 | 27 |
| 2N0A | Atomic-resolution structure of alpha-synuclein fibrils | SOLID-STATE NMR | n.a. | 23/03/2016 | 61 |
| 4RP7 | Structure of the amyloid-forming segment TIITLE from p53 (residues 253-258) | X-RAY DIFFRACTION | 1.576 Å | 13/01/2016 | 4 |
| 4RP6 | Structure of the amyloid-forming segment LTIITLE from p53 (residues 252-258) | X-RAY DIFFRACTION | 1.703 Å | 13/01/2016 | 5 |
| 5E5X | Structure of the amyloid forming peptide ANFLVH (residues 13-18) from islet amyloid polypeptide | X-RAY DIFFRACTION | 1.61 Å | 16/12/2015 | 4 |
| 5E5Z | Structure of the amyloid forming peptide LVHSSN (residues | X-RAY DIFFRACTION | 1.664 Å | 16/12/2015 | 4 |
| 5E5V | Structure of amyloid forming peptide NFGAILS (residues 22-28) from Islet Amyloid Polypeptide | X-RAY DIFFRACTION | 1.24 Å | 16/12/2015 | 4 |
| 5E61 | Structure of amyloid-forming peptide FGAILSS (residues 23-29) from islet amyloid polypeptide | X-RAY DIFFRACTION | 1.79 Å | 16/12/2015 | 5 |
| 4XFN | Structure of an Amyloid forming peptide AEVVFT from Human Transthyretin | X-RAY DIFFRACTION | 1.85 Å | 21/10/2015 | 4 |
| 4XFO | Structure of an amyloid-forming segment TAVVTN from human Transthyretin | X-RAY DIFFRACTION | 1.35 Å | 21/10/2015 | 4 |
| 5AEF | Electron cryo-microscopy of an Abeta(1-42)amyloid fibril | ELECTRON MICROSCOPY | 5 Å | 14/10/2015 | 25 |
| 4ZNN | MicroED structure of the segment, GVVHGVTTVA, from the A53T familial mutant of Parkinson's disease protein, alpha-synuclein residues 47-56 | ELECTRON CRYSTALLOGRAPHY | 1.41 Å | 09/09/2015 | 8 |
| 4RIK | Amyloid forming segment, AVVTGVTAV, from the NAC domain of Parkinson's disease protein alpha-synuclein, residues 69-77 | X-RAY DIFFRACTION | 1.854 Å | 26/08/2015 | 7 |
| 4RIL | Structure of the amyloid forming segment, GAVVTGVTAVA, from the NAC domain of Parkinson's disease protein alpha-synuclein, residues 68-78, determined by electron diffraction | ELECTRON CRYSTALLOGRAPHY | 1.43 Å | 26/08/2015 | 9 |
| 2N1E | MAX1 peptide fibril | SOLID-STATE NMR | n.a. | 29/07/2015 | 18 |
| 2MXU | 42-Residue Beta Amyloid Fibril | SOLID-STATE NMR | n.a. | 06/05/2015 | 30 |
| 2MPZ | Atomic model of the Abeta D23N "Iowa" mutant using solid-state NMR, EM and Rosetta modeling | SOLID-STATE NMR | n.a. | 22/04/2015 | 20 |
| 4R0P | Ifqins, an amyloid forming segment from human lysozyme spanning residues 56-61 | X-RAY DIFFRACTION | 1.52 Å | 17/12/2014 | 4 |
| 4R0U | Tgvtava, an amyloid forming segment from alpha synuclein, residues 72-78 | X-RAY DIFFRACTION | 1.38 Å | 17/12/2014 | 5 |
| 4R0W | Vvtgvta, an amyloid forming segment from alpha synuclein, residues 70-76 | X-RAY DIFFRACTION | 1.5 Å | 17/12/2014 | 5 |
| 2MVX | Atomic-resolution 3D structure of amyloid-beta fibrils: the Osaka mutation | SOLID-STATE NMR | n.a. | 26/11/2014 | 15 |
| 4NP8 | Structure of an amyloid forming peptide VQIVYK from the second repeat region of tau (alternate polymorph) | X-RAY DIFFRACTION | 1.51 Å | 18/12/2013 | 4 |
| 4NIN | DSVISLS segment 101-107 from Human Superoxide Dismutase | X-RAY DIFFRACTION | 1.402 Å | 04/12/2013 | 5 |
| 4NIO | GVTGIAQ segment 147-153 from Human Superoxide Dismutase with I149T mutation associated with a familial form of amyotrophic lateral sclerosis | X-RAY DIFFRACTION | 1.3 Å | 04/12/2013 | 5 |
| 4NIP | GVIGIAQ segment 147-153 from Human Superoxide Dismutase | X-RAY DIFFRACTION | 1.9 Å | 04/12/2013 | 5 |
| 2M5K | Atomic-resolution structure of a doublet cross-beta amyloid fibril | SOLID-STATE NMR ELECTRON MICROSCOPY | 12.7 Å | 04/12/2013 | 9 |
| 2M5M | Atomic-resolution structure of a triplet cross-beta amyloid fibril | SOLID-STATE NMR ELECTRON MICROSCOPY | 12.2 Å | 04/12/2013 | 9 |
| 2M4J | 40-residue beta-amyloid fibril derived from Alzheimer's disease brain | SOLID-STATE NMR | n.a. | 25/09/2013 | 25 |
| 2M5N | Atomic-resolution structure of a cross-beta protofilament | SOLID-STATE NMR | n.a. | 17/07/2013 | 6 |
| 4E0K | Crystal Structure of the amyloid-fibril forming peptide KDWSFY derived from human Beta 2 Microglobulin (58-63) | X-RAY DIFFRACTION | 0.97 Å | 19/12/2012 | 4 |
| 3SGS | Amyloid-related segment of alphaB-crystallin residues 95-100 | X-RAY DIFFRACTION | 1.703 Å | 21/03/2012 | 4 |
| 3SGO | Amyloid-related segment of alphaB-crystallin residues 90-100 | X-RAY DIFFRACTION | 2.557 Å | 21/03/2012 | 9 |
| 2Y3J | Structure of segment AIIGLM from the amyloid-beta peptide (Ab, residues 30-35) | X-RAY DIFFRACTION | 1.99 Å | 02/11/2011 | 4 |
| 2Y3K | Structure of segment MVGGVVIA from the amyloid-beta peptide (Ab, residues 35-42), alternate polymorph 1 | X-RAY DIFFRACTION | 1.90 Å | 02/11/2011 | 6 |
| 2Y3L | Structure of segment MVGGVVIA from the amyloid-beta peptide (Ab, residues 35-42), alternate polymorph 2 | X-RAY DIFFRACTION | 2.10 Å | 02/11/2011 | 6 |
| 2Y2A | Structure of segment KLVFFA from the amyloid-beta peptide (Ab, residues 16-21), alternate polymorph I | X-RAY DIFFRACTION | 1.91 Å | 26/10/2011 | 4 |
| 2Y29 | Structure of segment KLVFFA from the amyloid-beta peptide (Ab, residues 16-21), alternate polymorph III | X-RAY DIFFRACTION | 2.30 Å | 26/10/2011 | 4 |
| 3Q2X | Structure of an amyloid forming peptide NKGAII (residues 27-32) from amyloid beta | X-RAY DIFFRACTION | 1.451 Å | 12/10/2011 | 4 |
| 3OW9 | Structure of an amyloid forming peptide KLVFFA from amyloid beta, alternate polymorph II | X-RAY DIFFRACTION | 1.8 Å | 31/08/2011 | 4 |
| 3OVJ | Structure of an amyloid forming peptide KLVFFA from amyloid beta in complex with orange G | X-RAY DIFFRACTION | 1.8 Å | 06/07/2011 | 4 |
| 3OVL | Structure of an amyloid forming peptide VQIVYK from the TAU protein in complex with orange G | X-RAY DIFFRACTION | 1.81 Å | 06/07/2011 | 4 |
| 3PPD | GGVLVN segment from Human Prostatic Acid Phosphatase Residues 260-265, involved in Semen-Derived Enhancer of Viral Infection | X-RAY DIFFRACTION | 1.5 Å | 29/06/2011 | 4 |
| 2LBU | HADDOCK calculated model of Congo red bound to the HET-s amyloid | SOLID-STATE NMR | n.a. | 01/06/2011 | 57 |
| 3MD4 | Prion peptide | X-RAY DIFFRACTION | 1.15 Å | 25/05/2011 | 4 |
| 3NVG | MIHFGN segment 137-142 from mouse prion | X-RAY DIFFRACTION | 1.48 Å | 02/03/2011 | 4 |
| 3NVH | MIHFGND segment 137-143 from mouse prion | X-RAY DIFFRACTION | 1.61 Å | 02/03/2011 | 5 |
| 3NHC | GYMLGS segment 127-132 from human prion with M129 | X-RAY DIFFRACTION | 1.57 Å | 04/08/2010 | 4 |
| 2KJ3 | High-resolution structure of the HET-s(218-289) prion in its amyloid form obtained by solid-state NMR | SOLID-STATE NMR | n.a. | 02/06/2010 | 56 |
| 3HYD | LVEALYL peptide derived from human insulin chain B, residues 11-17 | X-RAY DIFFRACTION | 1 Å | 06/10/2009 | 5 |
| 3FPO | HSSNNF segment from Islet Amyloid Polypeptide (IAPP or Amylin) | X-RAY DIFFRACTION | 1.5 Å | 30/06/2009 | 4 |
| 3FR1 | NFLVHS segment from Islet Amyloid Polypeptide (IAPP or Amylin) | X-RAY DIFFRACTION | 1.85 Å | 30/06/2009 | 4 |
| 3FTR | Structure of an amyloid forming peptide SSTNVG from IAPP (alternate polymorph) | X-RAY DIFFRACTION | 1.61 Å | 30/06/2009 | 4 |
| 3FVA | NNQNTF segment from elk prion | X-RAY DIFFRACTION | 1.458 Å | 30/06/2009 | 4 |
| 3FTH | NFLVHSS segment from Islet Amyloid Polypeptide (IAPP or Amylin) | X-RAY DIFFRACTION | 1.84 Å | 30/06/2009 | 5 |
| 3FTK | NVGSNTY segment from Islet Amyloid Polypeptide (IAPP or Amylin), hydrated crystal form | X-RAY DIFFRACTION | 1.5 Å | 30/06/2009 | 5 |
| 3FTL | NVGSNTY segment from Islet Amyloid Polypeptide (IAPP or Amylin), dehydrated crystal form | X-RAY DIFFRACTION | 1.6 Å | 30/06/2009 | 5 |
| 3FOD | AILSST segment from Islet Amyloid Polypeptide | X-RAY DIFFRACTION | 1.4 Å | 19/05/2009 | 4 |
| 3DG1 | Segment SSTNVG derived from IAPP | X-RAY DIFFRACTION | 1.66 Å | 01/07/2008 | 4 |
| 3DGJ | NNFGAIL segment from Islet Amyloid Polypeptide (IAPP or amylin) | X-RAY DIFFRACTION | 1.8 Å | 01/07/2008 | 5 |
| 2ONV | Crystal Structure of the amyloid-fibril forming peptide GGVVIA derived from the Alzheimer's amyloid Abeta (Abeta37-42). | X-RAY DIFFRACTION | 1.61 Å | 06/02/2007 | 4 |
| 2ONW | Structure of SSTSSA, a fibril forming peptide from Bovine Pancreatic Ribonuclease (RNase A, residues 15-20) | X-RAY DIFFRACTION | 1.51 Å | 02/02/2007 | 4 |
| 2ONA | MVGGVV peptide derived from Alzheimer's A-beta, residues 35-40 | X-RAY DIFFRACTION | 2.03 Å | 30/01/2007 | 4 |
| 2ON9 | Structure of an amyloid forming peptide vqivyk from the repeat region of tau | X-RAY DIFFRACTION | 1.51 Å | 30/01/2007 | 4 |
| 2OKZ | MVGGVV peptide derived from Alzheimer's A-beta | X-RAY DIFFRACTION | 1.8 Å | 30/01/2007 | 4 |
| 2OMP | LYQLEN peptide derived from human insulin chain A, residues 13-18 | X-RAY DIFFRACTION | 1.90 Å | 30/01/2007 | 4 |
| 2OMQ | VEALYL peptide derived from human insulin chain B, residues 12-17 | X-RAY DIFFRACTION | 2.00 Å | 30/01/2007 | 4 |
| 2OMM | GNNQQNY peptide corresponding to residues 7-13 of yeast prion sup35 | X-RAY DIFFRACTION | 2.00 Å | 30/01/2007 | 5 |
| 1YJO | Structure of NNQQNY from yeast prion Sup35 with zinc acetate | X-RAY DIFFRACTION | 1.8 Å | 14/06/2005 | 4 |
| 1YJP | Structure of GNNQQNY from yeast prion Sup35 | X-RAY DIFFRACTION | 1.8 Å | 14/06/2005 | 5 |
| 2BFI | Molecular basis for amyloid fibril formation and stability | X-RAY DIFFRACTION | 1.1 Å | 06/01/2005 | 10 |
| 1RVS | STRUCTURE OF TRANSTHYRETIN IN AMYLOID FIBRILS DETERMINED BY SOLID-STATE MAGIC ANGLE SPINNING NMR | SOLID-STATE NMR | n.a. | 20/01/2004 | 9 |
